# The Neo-PLANET phase II trial of neoadjuvant camrelizumab plus concurrent chemoradiotherapy in locally advanced adenocarcinoma of stomach or gastroesophageal junction

**DOI:** 10.1038/s41467-022-34403-5

**Published:** 2022-11-10

**Authors:** Zhaoqing Tang, Yan Wang, Dan Liu, Xuefei Wang, Chen Xu, Yiyi Yu, Yuehong Cui, Cheng Tang, Qian Li, Jing Sun, Qian Zhang, Yuan Ji, Guifen Ma, Haojie Li, Zhenbin Shen, Kuntang Shen, Rongrong Zheng, Zhiguo Hou, Tianshu Liu, Jiping Wang, Yihong Sun

**Affiliations:** 1 Department of General Surgery, Zhongshan Hospital, Fudan University, Shanghai, China; 2 Gastric Cancer Center, Zhongshan Hospital, Fudan University, Shanghai, China; 3 Department of Medical Oncology, Zhongshan Hospital, Fudan University, Shanghai, China; 4 Department of Pathology, Zhongshan Hospital, Fudan University, Shanghai, China; 5 Department of Radiotherapy, Zhongshan Hospital, Fudan University, Shanghai, China; 6 Department of Medical Affairs, Jiangsu Hengrui Pharmaceuticals Co., Ltd., Shanghai, China; 7 Cancer Center, Zhongshan Hospital, Fudan University, Shanghai, China; 8 Division of Surgical Oncology, Brigham and Women’s Hospital, Harvard Medical School, Boston, MA USA

**Keywords:** Translational research, Gastric cancer, Cancer immunotherapy

## Abstract

The synergistic effect of neoadjuvant immunotherapy and chemoradiotherapy in gastric adenocarcinoma is unclear. This phase II trial (NCT03631615) investigated this neoadjuvant combination in locally advanced adenocarcinoma of stomach or gastroesophageal junction. Thirty-six patients received capecitabine 850 mg/m^2^ twice daily and simultaneous radiotherapy for 5 weeks, sandwiched by a 21-day cycle of oxaliplatin 130 mg/m^2^ (day 1) plus capecitabine 1000 mg/m^2^ twice daily (days 1–14), respectively, followed by surgery. Camrelizumab 200 mg (day 1) was given for 5 cycles since initiating chemotherapy. Primary endpoint was pathological complete response (pCR, ypT0) rate. Secondary endpoints included total pCR (tpCR, ypT0N0) rate, major pathological response (MPR, < 10% residual tumor cells) rate, margin-free (R0) resection rate, downstaging, progression-free survival (PFS), overall survival (OS), and safety. The pCR rate was 33.3% (95% CI, 18.6–51.0), meeting pre-specified endpoint. TpCR, MPR, and R0 resection rates were 33.3%, 44.4%, and 91.7%, respectively. Twenty-eight (77.8%) patients reached ypN0. Two-year PFS and OS rates were 66.9% and 76.1%, respectively. The most common grade 3–4 adverse event was decreased lymphocyte count (27 [75.0%]). Neoadjuvant camrelizumab plus concurrent chemoradiotherapy exhibits promising pathological response in patients with locally advanced gastric adenocarcinoma, with an acceptable safety profile.

## Abstract (web summary)

Surgery is an important part of treatment for gastric adenocarcinoma, however, the consensus on perioperative care is less clear. Here, the authors report the results of a phase II clinical trial investigating neoadjuvant camrelizumab with concurrent chemoradiotherapy, followed by surgery for the treatment of advanced gastric adenocarcinoma.

## Introduction

Gastric cancer is the third leading cause of death due to cancer worldwide^1^. Although the consensus on the surgical treatment has resulted in the improvement of curative effect during the past decades, controversies remained for the perioperative therapy of gastric cancer^2,3^, especially in the selection of the optimal neoadjuvant regimens. Only a small proportion (2–16%) of patients achieved pathological complete response (pCR) after neoadjuvant chemotherapy or chemoradiotherapy^4–7^, thus more effective regimens with multimodal therapy should be considered for patients with locally advanced gastric cancer.

Immunotherapy with anti-programmed cell death-1 (PD-1) or anti-programmed cell death-ligand 1 (PD-L1) antibody has demonstrated moderate efficacy in selected patients with advanced gastric adenocarcinoma^8–10^. The binding of PD-1 and its ligands can inhibit cytotoxic T-cell response, allowing tumor cells to evade immune detection^11^. As a result, blocking this interaction restores T-cell antitumor activity and leads to a long-lasting response in various types of tumors, with manageable toxicity^12^.

Given the success of immunotherapy plus chemotherapy in the first-line setting, neoadjuvant therapy containing PD-1/PD-L1 blockade has been investigated in gastric adenocarcinoma^13–18^, with a hypothesis of the promotion of systemic antitumor immunity, derived from the activation of tumor-specific T cells within the tumor microenvironment, and the enhancement of antigen presentation from the dendritic cell to the tumor-specific T cells^19^. Additionally, chemoradiotherapy might increase the expression of PD-1/PD-L1 in tumor cells and improve the efficacy of immunotherapy in PD-L1-negative or microsatellite-stable patients^20^. Hence, we hypothesized that neoadjuvant chemoradiotherapy combined with immunotherapy would result in synergistic antitumor activity and achieve more efficient therapeutic consequences.

In this phase II Neo-PLANET study, we show that camrelizumab (an anti-PD-1 antibody) plus concurrent chemoradiotherapy is effective and safe as neoadjuvant therapy in patients with resectable locally advanced adenocarcinoma of the stomach or gastroesophageal junction (GEJ), with a pCR rate of 33.3% (95% confidence interval [CI], 18.6–51.0) in the full analysis set (FAS).

## Results

### Patients’ characteristics and treatment

**Fig. 1:**
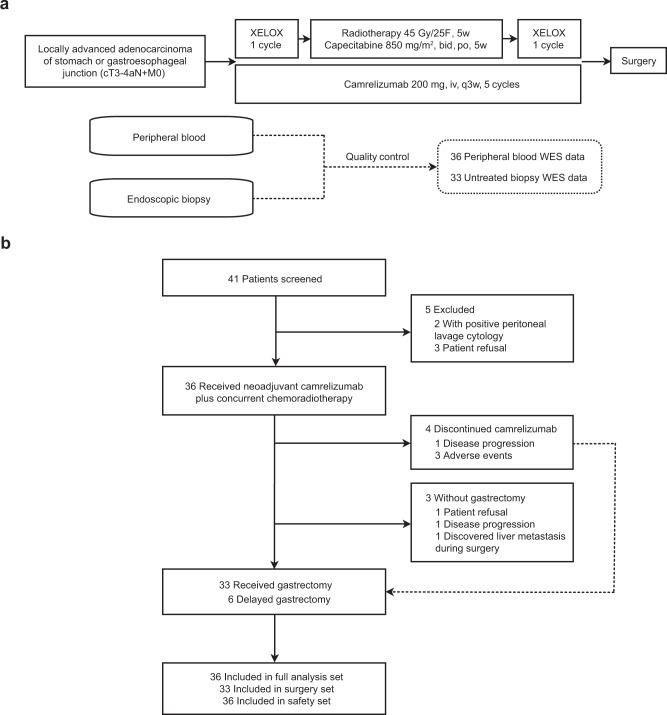
Study design and Consort diagram. **a** Trial schema and sample collection. **b** Consort diagram. XELOX, capecitabine and oxaliplatin, WES whole-exome sequencing.

Between September 14, 2018, and December 22, 2020, 41 patients were screened and 36 patients were enrolled (Fig. 1). Characteristics of 36 patients are listed in Table 1. All patients had lymph node involvement, 30 (83.3%) had T4a disease, and 19 (52.8%) had primary tumors located at GEJ. Twenty-five (69.4%) patients were assessed for PD-L1 expression, and nine had a combined positive score (CPS) of 1 or more. Twenty-seven (75%) patients were assessed for Epstein-Barr Virus (EBV) status, and only one had an EBV-positive tumor. Thirty-three (91.7%) patients were assessed for microsatellite instability (MSI) and tumor mutational burden (TMB) status; all the tumors were MSI-low/microsatellite stability (MSS), and one was TMB-high.

**Table 1:** Baseline characteristics of full analysis set (*N* = 36)

	Patients, No. (%)
Age (years), median (range)	65.5 (35–72)
**Sex**	
Male	28 (77.8)
Female	8 (22.2)
**ECOG performance status**	
0	36 (100)
**Primary tumor site**	
Gastroesophageal junction	19 (52.8)
Stomach	17 (47.2)
**Lauren’s classification**	
Intestinal type	19 (52.8)
Diffuse type	5 (13.9)
Mixed type	10 (27.8)
Unspecified type	2 (5.6)
**Clinical T stage**	
T3	6 (16.7)
T4a	30 (83.3)
**Clinical N stage**	
N +	36 (100)
**PD-L1 CPS**	
<1	16 (44.4)
≥1	9 (25.0)
≥5	8 (22.2)
≥10	7 (19.4)
UTA	11 (30.6)
**MSI**	
MSI-high	0
MSI-low/MSS	33 (91.7)
UTA	3 (8.3)
**TMB**	
TMB-low	32 (88.9)
TMB-high	1 (2.8)
UTA	3 (8.3)
**EBV status**	
Positive	1 (2.8)
Negative	26 (72.2)
UTA	9 (25.0)

*ECOG* Eastern Cooperative Oncology Group, *PD-L1* programmed cell death-ligand 1, *CPS* combined positive score, *MSI* microsatellite instability, *MSS* microsatellite stability, *TMB* tumor mutation burden, *EBV* Epstein-Barr virus, *UTA* unable to access.

All patients received neoadjuvant therapy and thus were included in the FAS; 32 patients completed neoadjuvant therapy as planned, while one discontinued camrelizumab due to disease progression and three discontinued camrelizumab due to adverse events (AEs): one had neuritis presented with headache and tongue movement disorder, one had myositis and myocarditis with impaired heart function, and one had severe reactive cutaneous capillary endothelial proliferation widely distributed throughout the body. Thirty-three patients underwent total gastrectomy and D2 lymph node dissection, including 30 with open surgery and three with laparoscopic surgery. One patient with open surgery also had a combined splenectomy. Six (18.2%) of 33 patients had delayed surgery: five due to immune-related AEs (irAEs) and one due to non-irAEs (prolonged grade 2 nausea and vomiting). The median operative time was 186 min (range, 140–259). The median intraoperative blood loss was 100 mL (range, 20–400). The median number of lymph nodes resected was 28 (range, 6–51). The median length of postoperative hospital stay was 8 days (range, 6–20). Three patients did not undergo gastrectomy because one had progressive disease, one refused surgery, and one was deemed uncurable due to liver metastases during surgical exploration.

### Efficacy

**Fig. 2:**
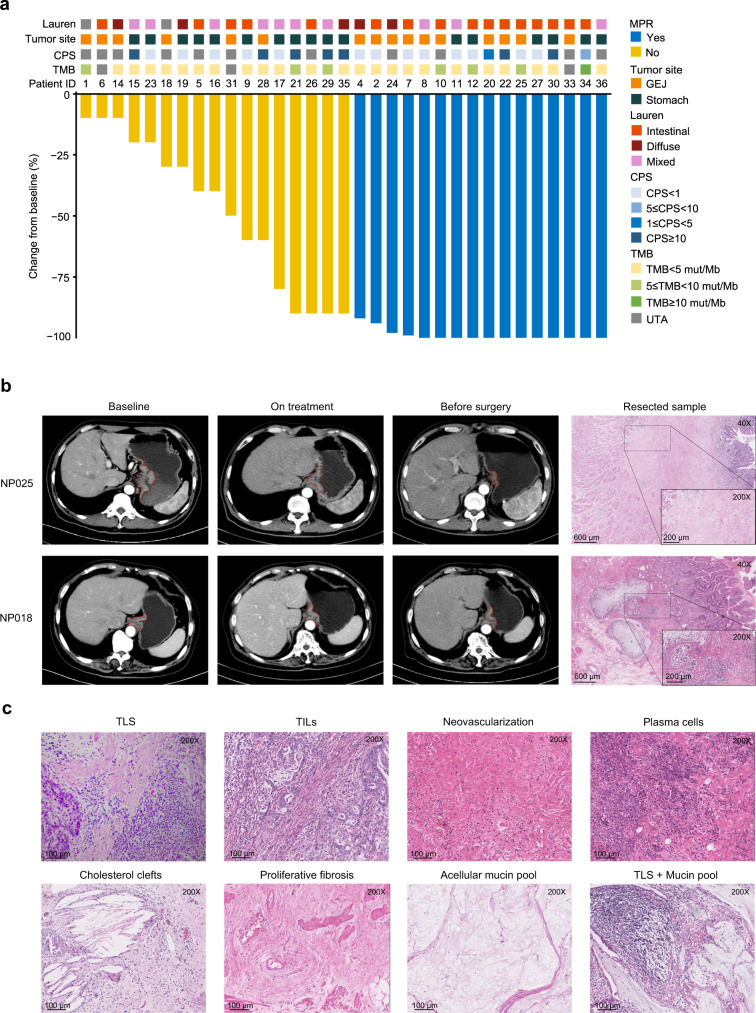
Tumor response to neoadjuvant therapy. **a** Waterfall plot of tumor regression by pathology (*n* = 33). **b** Representative radiological and pathological images (HE staining, ×40, ×200) from responsive (NP025) and nonresponsive (NP018) patients. HE staining was performed one time in 33 independent samples with similar results. **c** Representative pathological features (HE staining, ×200) after immunotherapy. HE staining was performed one time in 33 independent samples with similar results. CPS combined positive score, UTA unable to access, GEJ gastroesophageal junction, MPR major pathologic response, HE hematoxylin and eosin, TLS tertiary lymphoid structures, TILs tumor-infiltrating lymphocytes. Source data are provided as a Source Data file.

In the FAS, 12 of 36 patients had pathological downstaging to ypT0N0, with both pCR (primary endpoint) and total pCR (tpCR; secondary endpoint) rates of 33.3% (95% CI, 18.6–51.0). In the surgery set, both pCR and tpCR rates were 36.4% (95% CI, 20.4–54.9). The major pathological response (MPR; secondary endpoint) rate was 44.4% (95% CI, 27.9–61.9) in the FAS and 48.5% (95% CI, 30.8–66.5) in the surgery set (Table 2). Four of eight patients with grade 2 tumor regression had just 10% residual tumor cells (Fig. 2a). Representative changes in tumor size by imaging examinations and postoperative pathological images from one patient with pCR (NP025) and another patient with a minor pathological response (NP018; grade 3 tumor regression) are shown in Fig. 2b. Representative pathological features^21^ after immunotherapy are shown in Fig. 2c. The downstaging (secondary endpoint) results showed that 28 (77.8%) of 36 patients reached ypN0 (Supplementary Table 1). Patients with intestinal type had higher pCR rate than those with other types (56.3% [9/16] vs. 17.6% [3/17], *P* = 0.032). The pCR rates for patients with tumor at GEJ and stomach were 37.5% (6/16) and 35.3% (6/17; *P* > 0.999; Supplementary Table 2), respectively. Of five patients with delayed surgery due to irAEs, four achieved pCR, and one had grade 3 tumor regression (80% residual tumor cells). The margin-free (R0) resection rate (secondary endpoint) was 91.7% in the FAS and 100% in the surgery set.

**Table 2:** Pathological response

	Full analysis set (*n* = 36)	Surgery set (*n* = 33)
Becker’s TRG		
1a	12 (33.3)	12 (36.4)
1b	4 (11.1)	4 (12.1)
2	8 (22.2)	8 (24.2)
3	9 (25.0)	9 (27.3)
UTA	3 (8.3)	0
pCR rate	12 (33.3)	12 (36.4)
95% CI	18.6–51.0	20.4–54.9
tpCR rate	12 (33.3)	12 (36.4)
95% CI	18.6–51.0	20.4–54.9
MPR rate	16 (44.4)	16 (48.5)
95% CI	27.9–61.9	30.8–66.5

Data were *n* (%) unless otherwise stated.

*TRG* tumor regression grade, *pCR* pathological complete response, *UTA* unable to access, *tpCR* total pathological complete response, *MPR* major pathological response, *CI* confidence interval.

**Fig. 3:**
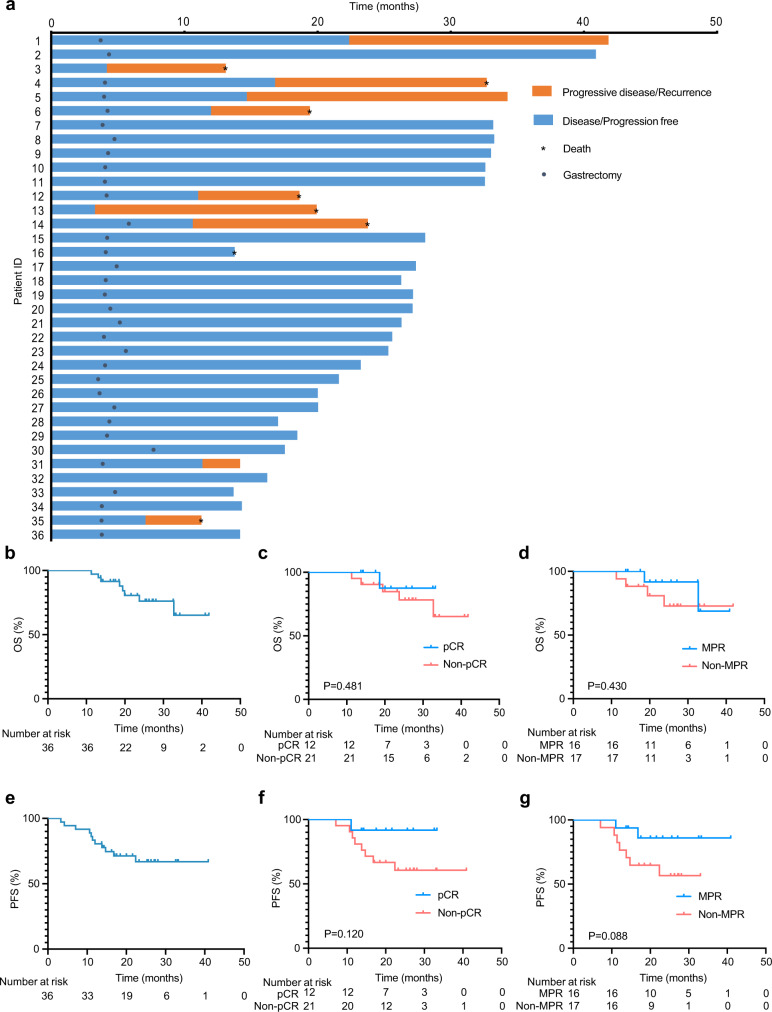
Follow-up and survival. **a** Swimming plot showing events during treatment and follow-up (*n* = 36). **b** Kaplan–Meier curves for overall survival in the full analysis set. **c** Kaplan–Meier curves for overall survival in patients with or without pCR. A two-sided log-rank test was used to determine the statistical significance between subgroups. **d** Kaplan–Meier curves for overall survival in patients with or without MPR. A two-sided log-rank test was used to determine the statistical significance between subgroups. **e** Kaplan–Meier curves for progression-free survival in the full analysis set. **f** Kaplan–Meier curves for progression-free survival in patients with or without pCR. A two-sided log-rank test was used to determine the statistical significance between subgroups. **g** Kaplan–Meier curves for progression-free survival in patients with or without MPR. A two-sided log-rank test was used to determine the statistical significance between subgroups. pCR pathological complete response, MPR major pathological response. Source data are provided as a Source Data file.

By the data cutoff date on February 20, 2022, the median follow-up time was 26.3 months (range, 11.3–41.8). Both median progression-free survival (PFS; secondary endpoint) and overall survival (OS; secondary endpoint) were not reached. The post-hoc analyses showed that the 2-year PFS rate was 66.9% and the 2-year OS rate was 76.1% (Fig. 3b–g). The 2-year PFS and OS rates were 60.0% and 73.1% in patients with tumors at GEJ, and 75.5 and 80.2% in patients with tumors at the stomach (Supplementary Fig. 1), respectively. As demonstrated in Fig. 3a, eight patients who underwent gastrectomy experienced recurrence, and five of them died. Two patients who suffered tumor progression during neoadjuvant therapy died of progressive disease. One patient free from progression died by accident and no recurrence was determined.

### Safety

Treatment-emergent AEs (TEAEs) of any grade occurred in all patients during neoadjuvant therapy. Grade ≥3 TEAEs occurred in 28 (77.8%) patients. The most common grade 3 or 4 TEAEs were decreased lymphocyte count (27 [75.0%]) and decreased white blood cell count (two [5.6%]; Table 3). IrAEs occurred in 31 (86.1%) patients, but most were grade 1–2. The most common irAEs were reactive cutaneous capillary endothelial proliferation (25 [69.4%]), thyroid dysfunction (six [16.7%]), hyperglycemia (six [16.7%]), and pruritus (five [13.9%]; Table 4). No AEs leading to death occurred.

**Table 3:** Treatment-emergent adverse events occurring in 5% or more of patients (*N* = 36)

	No. (%)				
	Any grade	Grade 1	Grade 2	Grade 3	Grade 4
Any events	36 (100)	36 (100)	28 (77.8)	25 (69.4)	6 (16.7)
**General disorders**					
Weight loss	5 (13.9)	2 (5.6)	3 (8.3)	0	0
Cough	2 (5.6)	2 (5.6)	0	0	0
Urinary tract infection	2 (5.6)	1 (2.8)	1 (2.8)	0	0
**Hematological**					
Lymphocyte count decreased	35 (97.2)	3 (8.3)	5 (13.9)	24 (66.7)	3 (8.3)
White blood cell decreased	26 (72.2)	9 (25.0)	15 (41.7)	2 (5.6)	0
Anemia	25 (69.4)	21 (58.3)	4 (11.1)	0	0
Platelet count decreased	19 (52.8)	13 (36.1)	6 (16.7)	0	0
Neutropenia	17 (47.2)	9 (25.0)	8 (22.2)	0	0
**Biochemistry**					
Hypocalcaemia	6 (16.7)	5 (13.9)	1 (2.8)	0	0
Hypokalemia	3 (8.3)	3 (8.3)	0	0	0
Hyponatremia	3 (8.3)	2 (5.6)	1 (2.8)	0	0
Hypoalbuminemia	3 (8.3)	2 (5.6)	1 (2.8)	0	0
Hyperuricemia	3 (8.3)	3 (8.3)	0	0	0
Blood bilirubin increased	2 (5.6)	1 (2.8)	1 (2.8)	0	0
**Gastrointestinal disorders**					
Vomiting	16 (44.4)	11 (30.6)	5 (13.9)	0	0
Nausea	15 (41.7)	12 (33.3)	2 (5.6)	1 (2.8)	0
Loss of appetite	3 (8.3)	2 (5.6)	1 (2.8)	0	0
Diarrhea	2 (5.6)	2 (5.6)	0	0	0
Abdominal distension	2 (5.6)	2 (5.6)	0	0	0
Constipation	2 (5.6)	2 (5.6)	0	0	0

No grade 5 treatment-emergent adverse events occurred.

**Table 4:** All immune-related adverse events (*N* = 36)

	No. (%)				
	Any grade	Grade 1	Grade 2	Grade 3	Grade 4
Any events	31 (86.1)	30 (83.3)	2 (5.6)	2 (5.6)	3 (8.3)
**Cutaneous**					
Reactive cutaneous capillary endothelial proliferation	25 (69.4)	24 (66.7)	0	1 (2.8)	0
Pruritus	5 (13.9)	5 (13.9)	0	0	0
Rash	1 (2.8)	1 (2.8)	0	0	0
**Endocrine**					
Thyroid dysfunction	6 (16.7)	4 (11.1)	2 (5.6)	0	0
Hyperglycemia	6 (16.7)	5 (13.9)	0	1 (2.8)	0
**Others**					
Immune-related myocarditis	2 (5.6)	0	0	0	2 (5.6)
Immune-related hepatitis	1 (2.8)	0	0	0	1 (2.8)
Immune-related pneumonia	1 (2.8)	0	0	0	1 (2.8)
Immune-related myositis	1 (2.8)	0	0	0	1 (2.8)
Immune-related neuritis	1 (2.8)	0	0	0	1 (2.8)

No grade 5 immune-related adverse events occurred.

Among 33 patients with surgery, surgical complications occurred in 13 (39.4%) patients. The most frequent grade II or III surgical complications were pleural effusion (four [12.1%]), atelectasis (three [9.1%]), and intra-abdominal infection (three [9.1%]; Supplementary Table 3). One patient with intra-abdominal bleeding and one patient with intra-abdominal infection suffered reoperation, both recovered eventually. No grade IV or V surgical complications occurred. No patients had surgical complications leading to readmission within 30 days.

### Biomarkers

The pCR rate in PD-L1-positive tumors (defined as CPS at a cutoff point of 1, 5, or 10, respectively) was not significantly higher than in PD-L1-negative tumors (Supplementary Table 4). Analysis of somatic mutation by whole-exome sequencing (WES) of treatment specimens showed a significantly higher pCR rate in patients with pretreatment TMB ≥ median level (4.04 mutation/Mb) than those with TMB < median level. All six patients with intestinal type and TMB ≥ median level achieved pCR (Supplementary Table 4). For MSI and EBV, no tumor was identified as MSI-high by WES and only one tumor was found EBV-positive, so we could not provide information that the MSI-high or EBV-positive was related to tumor response in this study. The only patient with an EBV-positive tumor had grade 3 tumor regression (80% residual tumor cells). The PD-L1 density confirmed by immunohistochemistry (IHC) significantly increased after neoadjuvant therapy (Supplementary Fig. 2).

## Discussion

This Neo-PLANET study provided evidence for the combination of anti-PD-1 antibody camrelizumab and concurrent chemoradiotherapy as neoadjuvant therapy for locally advanced gastric adenocarcinoma, which resulted in a pCR rate of 33.3% (95% CI, 18.6–51.0), MPR rate of 44.4% (95% CI, 27.9–61.9), and R0 resection rate of 91.7% in 36 patients with resectable T3-4N + M0 adenocarcinoma of stomach or GEJ.

The effect of neoadjuvant immunotherapy has been evaluated in many solid tumors^19^. Camrelizumab as a component of neoadjuvant therapy has also been investigated in locally advanced gastric adenocarcinoma^16,17^ and esophageal squamous cell carcinoma^22–24^. In our study, neoadjuvant camrelizumab plus concurrent chemoradiotherapy exhibited a pCR rate of 33.3% in the FAS and 36.4% in the surgery set, higher than camrelizumab plus chemotherapy (9.5 and 11.5% in the surgery set)^16,17^, sintilimab plus chemotherapy (19.4 and 18.8% in the surgery set)^13,14^, toripalimab plus chemotherapy (25.0% in the surgery set)^15^ in patients with locally advanced gastric or GEJ adenocarcinoma. Recently, the randomized DANTE trial reported the interim results of neoadjuvant atezolizumab plus chemotherapy in 146 patients with resectable esophagogastric adenocarcinoma. Compared with our study, DANTE also reported a lower pCR rate (25% in the FAS), but a slightly higher MPR rate (49% in the FAS)^18^. Considering the single-arm design of our study with small sample size, a large-scale randomized controlled trial is warranted to validate camrelizumab plus concurrent chemoradiotherapy in patients with gastric cancer.

Chemoradiotherapy has become the standard neoadjuvant therapy in patients with resectable esophageal or GEJ cancer based on the results of the CROSS study^25^, while its use in gastric cancer is still being discussed. The phase III POET study demonstrated that neoadjuvant chemoradiotherapy could result in a better pCR rate (15.6 vs. 2.0% in the surgery population) compared with chemotherapy in patients with T3-4NXM0 GEJ adenocarcinoma^4^. The phase III TOPGEAR^26^ and PREACT^27^ trials comparing neoadjuvant chemoradiotherapy with chemotherapy in patients with gastric or GEJ adenocarcinoma are ongoing. Ajani et al. conducted three single-arm phase II trials to investigate neoadjuvant chemoradiotherapy in gastric adenocarcinoma, with a pCR rate of 19.5–30.3% in the FAS^28–30^. The relatively high pCR rate might be attributed to the clinical stage at baseline (6.1–18.6% had T1-2, none had T4, and 37.2–40.9% had N0 disease)^28–30^. Liu et al. conducted a phase II trial of neoadjuvant chemoradiotherapy in patients with locally advanced gastric or GEJ adenocarcinoma^31^, with similar population and perioperative treatment strategy to our study. The major discrepancies were the use of different chemotherapy regimens between studies (S-1 plus oxaliplatin in the study by Liu et al.^31^ versus XELOX in our study) and the addition of neoadjuvant camrelizumab in our study. Despite the use of different chemotherapy regimens, the increase in pCR rate from 13.9^31^ to 33.3% might have supported the combination of PD-1 inhibitors and concurrent chemoradiotherapy. The 2-year PFS rate (66.9%) in our study was also higher than the study by Liu et al. (47%)^31^, and perioperative standard FLOT regimen (fluorouracil plus leucovorin, oxaliplatin and docetaxel; ~55–60%)^7,32^. Neoadjuvant PD-1 inhibitor plus concurrent chemoradiotherapy might delay disease progression in gastric or GEJ adenocarcinoma. However, these indirect comparisons should be interpreted with caution, considering the small-sample results and different clinical settings across studies.

Our study did not show any predictive value of PD-L1 expression to tumor response, even a trend. In the PERFECT study, there was a numerically higher proportion of responders (grade 1–2 tumor regression) with CPS ≥10 compared with nonresponders, but also without statistical significance (62 vs. 30%, *P* = 0.069)^33^. One of the possible explanations is that the synergistic treatment effect of combining radiotherapy with immunotherapy overrode PD-L1 predictivity. It should also be noted that both studies might lack statistical power due to the small sample size. The predictive role of PD-L1 expression for neoadjuvant immunotherapy in patients with gastric or GEJ adenocarcinoma needs further investigation.

The AEs with neoadjuvant camrelizumab plus XELOX chemotherapy (oxaliplatin and capecitabine) and concurrent radiotherapy were similar to the safety profile in the previous reports^16,17,34,35^. No new safety signals were identified. The most common grade 3 or 4 TEAE was decreased lymphocyte count, which could recover after dose reduction or delay. The other AEs and surgical complications were all manageable. No AEs or surgical complications leading to death occurred. On the other hand, five (15.2%) patients had delayed surgery due to irAEs, but four of them achieved pCR. This indicated that the extended interval between neoadjuvant immunotherapy and surgery might not affect the pathological benefit. Redefinition of delayed surgery and longer permissible intervals could be considered when immunotherapy was added to neoadjuvant therapy. Given the positive results in phase III CheckMate 577 study of adjuvant nivolumab in esophageal or GEJ cancer^36^, the timing of immunotherapy in the perioperative setting and the role of adjuvant camrelizumab could be further investigated in future trials.

There are some limitations in this study. The single-arm, single-center design might lead to potential selection bias. The sample size was relatively small, and the subgroup analyses might be underpowered. In addition, 30.6% of patients did not have available data on PD-L1 expression because of missing or inadequate biopsy specimens. The median PFS and OS are not mature yet, which will be reported in our future reports.

In conclusion, neoadjuvant camrelizumab plus concurrent chemoradiotherapy exhibits promising pathological responses in patients with resectable locally advanced adenocarcinoma of the stomach or GEJ, with an acceptable safety profile. This study supplemented the evidence on a potential neoadjuvant combination of anti-PD-1 antibody and chemoradiotherapy in this population. Survival follow-up needs to be continued.

## Methods

This study was conducted in accordance with the Declaration of Helsinki and Good Clinical Practice, and approved by the Ethics Committee of Zhongshan Hospital, Fudan University. Written informed consent was obtained from each patient. Patients received the study treatment free of charge without other compensations. The study was preregistered with ClinicalTrials.gov on August 15, 2018 (identifier: NCT03631615).

### Study design and participants

In this single-arm phase II clinical trial, patients were enrolled from Zhongshan Hospital, Fudan University between September 14, 2018, and December 22, 2020. Eligible patients were aged 18–75 years; had Eastern Cooperative Oncology Group (ECOG) performance status of 0–1; had histological confirmed adenocarcinoma located at stomach or GEJ (Siewert type II or III); had clinical stage T3-4aN + M0 by endoscopic ultrasound or enhanced computed tomography (CT)/magnetic resonance imaging (MRI) according to the eighth edition of the American Joint Committee on Cancer TNM staging system^37^; had treatment-naïve disease with the possibility for radical surgery; did not have severe comorbidity that might lead to expected survival less than 5 years, and had adequate organ function. All patients underwent staging laparoscopy to exclude peritoneal metastasis before enrollment. The study protocol listing the full eligibility criteria is provided as Supplementary Note 1 in the Supplementary Information file.

### Procedures

Patients received induction chemotherapy with oxaliplatin 130 mg/m^2^ on day 1 and capecitabine 1000 mg/m^2^ twice per day on days 1–14 (XELOX regimen) for a 21-day cycle. Five weeks of concurrent chemoradiotherapy was performed within one week after the completion of induction chemotherapy. Intensity-modulated radiotherapy was performed using a VersaHD accelerator (Elekta, Stockholm). Patients were fixed in a supine position by a vacuum-forming mold and scanned with CT. Images were transferred to the radiotherapy planning system (Monaco, Elekta CMS, Maryland Heights, MO, USA and Pinnacle3, Philips Medical Systems, Cleveland, Inc.). The thickness of each scan layer was 3 mm. Gross tumor volume (GTV) consisting of the primary tumor and visible metastatic lymph nodes was delineated based on CT/MRI and gastroscope. Clinical target volume (CTV) comprised of GTV and elective regional lymph nodes at high risk, and planning target volume (PTV) covered CTV and 5–10 mm beyond its margin. A dose-volume histogram was used to optimize the dose-distribution plan. The total irradiation dose of intensity-modulated radiotherapy was 45 Gy for PTV, delivered in 25 fractions, with 1.8 Gy on days 1–5 each week. Simultaneous capecitabine (850 mg/m^2^ twice per day) was given on each day of radiotherapy. Patients received consolidation chemotherapy with XELOX regimen for a cycle at 2–3 weeks after concurrent chemoradiotherapy and were reevaluated 1–3 weeks later. Camrelizumab 200 mg once every 3 weeks was given five times since the initiation of induction chemotherapy until 3 weeks before surgery. Patients with resectable disease proceeded to gastrectomy and D2 lymph node dissection, the extent of resection was according to Japanese gastric cancer treatment guidelines (version 4)^38^. Adjuvant chemotherapy with the XELOX regimen was recommended for four cycles at 4–6 weeks after surgery.

Dose reduction for capecitabine and oxaliplatin and delay for the three study drugs and radiotherapy were allowed according to the AEs during the neoadjuvant therapy period. The details of dose adjustment are described in the study protocol, provided in Supplementary Note 1. If disease progression, distant metastasis, or intolerable toxicity occurred before surgery, patients would discontinue the study treatment and switch to other appropriate treatment, with follow-up for PFS and OS.

Radiological assessment was performed by CT/MRI according to the Response Evaluation Criteria In Solid Tumors version 1.1 at baseline, after the completion of concurrent chemoradiotherapy, before surgery, every 6 months after surgery until 3 years, and annually thereafter. The pathological response was assessed according to Becker’s tumor regression grading system^39^. For the primary tumor, if the macroscopic tumor was identifiable or the area of the stomach with scarring could indicate the site of the previous tumor (tumor bed), the tumor or tumor bed would be cross-sectioned serially at 0.5 cm intervals. If the macroscopic tumor was unidentifiable, the tumor bed would be indicated by baseline gastroscopy and CT, and cross-sectioned serially at 0.5 cm intervals. For lymph nodes, surgeons separated the lymph nodes from the specimen and grouped them into separate stations before formalin-fixation. If the short diameter of a lymph node was more than 1 cm, this lymph node would be divided into two and embedded separately. Other lymph nodes were embedded with a maximum of 4 lymph nodes in one cage. The lymph nodes were cross-sectioned serially at 0.2–0.3 cm intervals. These tissue sections were stained with hematoxylin and eosin (HE) staining, elastic von Gieson staining, and periodic acid–Schiff (PAS) staining. The von Gieson staining was used to distinguish between tumor desmoplasia and scarring as a result of chemotherapy, and the PAS staining was used to help distinguish signet ring cells from histiocytes. IHC for cytokeratins was used when epithelial cells were unidentifiable. If no residual tumor cells were detected, additional three-step sectioning would be performed to confirm pCR.

AEs since the initiation of neoadjuvant therapy until 28 days after the last dose of neoadjuvant therapy were graded according to the National Cancer Institute Common Terminology Criteria for Adverse Events version 4.03. Intraoperative and postoperative surgical complications were graded according to the Clavien-Dindo classification^40^.

### Endpoints

The primary endpoint was pCR (ypT0) rate, defined as the proportion of patients with grade 1a (no residual tumor) regression at the primary tumor. Secondary endpoints included tpCR (ypT0N0) rate (defined as the proportion of patients with grade 1a regression at both primary tumor and lymph nodes), MPR rate (defined as the proportion of patients with grade 1a and 1b [<10% residual tumor cells] regression at primary tumor), R0 resection rate, downstaging, PFS (defined as the time from the initiation of neoadjuvant therapy to disease recurrence, progression, or any-cause death), OS (defined as the time from the initiation of neoadjuvant therapy to any-cause death), and safety.

### Biomarker analysis

Tumor specimens from pretreatment gastroscopy biopsy and surgical resection were collected for biomarker analysis. The PD-L1 expression level was measured by the 22C3 pharmDx kit (Agilent Technologies, catalog number M3653, clone number 22C3, 1:50 dilution) on the Dako Autostainer Link 48 system (Agilent Technologies). CPS was used to characterize the PD-L1 expression, calculated as the number of all PD-L1-positive cells (tumor cells, lymphocytes, and macrophages) divided by the number of all tumor cells ×100. EBV status of the tumor was determined on available formalin-fixed paraffin-embedded (FFPE) tissue sections by EBV-encoded small RNA in situ hybridization kit (Talent biomedical, catalog number CISH-EBER-100) according to the manufacturer’s instructions.

IHC slides were scanned and digitalized using a Scanscope XT system (Aperio/Leica Technologies). Single-staining IHC quantification analysis was performed by the pathologist using HALO 3.2 software (Indica Labs). The number of marker-positive cells for each analysis area were calculated and expressed as density (number of positive cells/mm^2^).

TMB and MSI status were determined by WES. Tumor DNA was isolated from FFPE sections of specimens. Maxwell 16 FFPE Plus LEV DNA Purification Kit (Promega, catalog number AS1135) was used to extract FFPE DNA after FFPE sample sections were scalpeled and deparaffinized. Total DNA from tissues (or blood) was extracted using DNeasy 96 Blood & Tissue Kit (Qiagen, catalog number 69504) or Blood Kit (Qiagen, catalog number 51104) according to the manufacturer’s instructions. About 300 ng high-quality genomic DNA concentrations (OD260/280 = 1.8–2.0) were sheared to target of 150–200 bp average size with Covaris LE220 Sonicator (Covaris) and were prepared into DNA libraries using SureselectXT reagent kit (Agilent, catalog number G9611A). A paired-end DNA sequencing library was prepared via end-repair, purifying, A-tailing, paired-end adapter ligation, and amplification. DNA concentration of prepared libraries was measured using a Qubit 3.0 fluorometer (Thermo Fisher Scientific) and the size distribution of the resulting sequencing libraries was analyzed using Agilent Bioanalyzer 4200 (Agilent). Paired-end sequencing is performed using an Illumina NovaSeq 6000 system following Illumina-provided protocols for 2 × 150 paired-end sequencing. Mutations were analyzed using Genome variant caller (GVC) software with default parameters and were annotated using VEP software (Ensembl Variant Effect Predictor, release_100.2)^41^. TMB was defined as the number of nonsynonymous variations with an allele frequency of at least 5% in the captured coding region, and the mutational type contained single nucleotide variants and indels. TMB-high was defined as ≥10 mutation/Mb. MSIsensor algorithm^42^, which identifies the percentage of microsatellite loci that are unstable in the tumor genome compared to its matched normal, was used to analyze the MSI status of each sample. MSI status was classified as MSI-high if the proportion of MSI exceeds 10%.

### Statistics and reproducibility

This was a single-arm study, and no randomization was used. Biomarker analyses and HE staining of pathological images were performed one time in each independent sample. No data were excluded from the statistical analyses. The investigators were not blinded to allocation during the study period. The pathological response was assessed by independent pathologists who were blinded to patient information.

Simon’s min-max two-stage design was adopted to calculate the sample size. We assumed that the pCR rate with neoadjuvant chemoradiotherapy as historical control was 16%^4,31,43^. The addition of camrelizumab to chemoradiotherapy would improve the pCR rate to 35%, with one-sided α of 5% and power of over 80%. In the first stage, if three or more of the 15 patients achieved pCR, another 21 would be accrued to the second stage. If ten or more of the 36 patients achieved pCR, the study treatment would be deemed worthy of future study.

The FAS included all patients who received at least one dose of study treatment. The surgery set included all patients who underwent gastrectomy. Efficacy was analyzed in the FAS and surgery set. AEs during neoadjuvant therapy were analyzed in the safety set, including all patients who received at least one dose of study treatment. Surgical complications were analyzed in the surgery set. Continuous data were expressed as median (range), and categorical data were expressed as frequency (percentage). The 95% CIs of pCR, tpCR, and MPR rates were calculated using the Clopper-Pearson method. PFS and OS were estimated using the Kaplan–Meier method, and post-hoc analyses were performed to calculate 2-year PFS and OS rates. Comparisons of pCR rate in different subgroups were performed using Fisher Exact test, and PFS and OS were compared using a log-rank test. Subgroups were divided by a pathological response (pCR vs. non-pCR; MPR vs. non-MPR), Lauren’s classification (intestinal type vs. diffuse type vs. mixed type vs. unspecified type; intestinal type vs. others), primary tumor site (GEJ vs. stomach), baseline CPS (≥1 vs. <1; ≥5 vs. <5; ≥10 vs. <10), and baseline TMB level (≥median vs. <median). A comparison of PD-L1 density before and after neoadjuvant therapy was performed using paired *t*-test. Two-sided *P* < 0.05 was considered statistically significant. Data were collected using Microsoft Office Excel 2010. Data analysis was performed using R software (version 4.0.3).

## Supplementary information

Supplementary Information

## Data Availability

The raw WES data generated in this study have been deposited in the Genome Sequence Archive^44^ in National Genomics Data Center, China National Center for Bioinformation^45^ under accession code HRA003201. The raw sequencing data are available under restricted access due to data privacy laws. Data are available on request sharing by sending requests to the corresponding author Yihong Sun (sun.yihong@zs-hospital.sh.cn), which will need the approval of the institutional ethical committees. Access can be obtained by completing the application form via GSA-Human System. For detailed guidance on making the data access request, see GSA-Human_Request_Guide_for_Users [https://ngdc.cncb.ac.cn/gsa-human/document/GSA-Human_Request_Guide_for_Users_us.pdf]. The approximate response time for accession requests is about 2 weeks. Clinical data were not publicly available due to involving patient privacy, but can be accessed from the corresponding author Yihong Sun (Email: sun.yihong@zs-hospital.sh.cn), upon request for 3 years; individual de-identified patient data will be shared for clinical study analyses. The remaining data are available in the manuscript, Supplementary Information, or Source Data file. The study protocol is provided in the Supplementary Information file. Source data are provided with this paper.

## Source data

Source Data
