# Supplementary material for: The Neo-PLANET phase II trial of neoadjuvant camrelizumab plus concurrent chemoradiotherapy in locally advanced adenocarcinoma of stomach or gastroesophageal junction
Source: Nat Commun. 2022 Nov 10;13:6807. doi: 10.1038/s41467-022-34403-5 (PMC9649722; doi:10.1038/s41467-022-34403-5)
Supplement: Supplementary file 1 — Supplementary Information [file 41467_2022_34403_MOESM1_ESM.pdf]

Table of contents

Supplementary Table 1. Downstaging in the surgery set 2

Supplementary Table 2. Comparison of pCR rate among subgroups by Lauren's classification or primary tumor site in the surgery set 3

Supplementary Table 3. Surgical complications 4

Supplementary Table 4. Relationship between PD-L1 or TMB at baseline and pathological response in the surgery set 5

Supplementary Figure 1. Survival analysis in subgroup by tumor site 6

Supplementary Figure 2. Comparison of PD-L1 density before and after neoadjuvant therapy 7

Supplementary Note 1. Study protocol 8

Supplementary Table 1. Downstaging in the surgery set (N = 33).

	No. (%)	
	Clinical stage at baseline	Pathological stage after surgery
T stage		
T0	0	12 (36.4)
T1b	0	4 (12.1)
T2	0	5 (15.2)
T3	6 (18.2)	8 (24.2)
T4a	27 (81.8)	4 (12.1)
N stage		
N0	0	28 (84.9)
N+	33 (100)	0
N1	0	3 (9.1)
N2	0	1 (3.0)
N3b	0	1 (3.0)
T downstaged		28 (84.9)
N downstaged		28 (84.9)

Supplementary Table 2. Comparison of pCR rate among subgroups by Lauren's classification or primary tumor site in the surgery set (N = 33).

	pCR	Non-pCR	P
Lauren's classification			0.081
Intestinal type (n = 16)	9 (56.3)	7 (43.7)	
Diffuse type (n = 5)	0	5 (100)	
Mixed type (n = 10)	3 (30.0)	7 (70.0)	
Unspecified type (n = 2)	0	2 (100)	
Lauren's classification			0.032
Intestinal type (n = 16)	9 (56.3)	7 (43.7)	
Others (n = 17)	3 (17.6)	14 (82.4)	
Primary tumor site			>0.999
Gastroesophageal junction (n = 16)	6 (37.5)	10 (62.5)	
Stomach (n = 17)	6 (35.3)	11 (64.7)	

Data are n (%). Two-sided Fisher Exact test was used to determine statistical significance among subgroups. pCR, pathological complete response.

Supplementary Table 3. Surgical complications (N = 33).

	No. (%)			
	Any grade	Grade I	Grade II	Grade III[a]
Any event	13 (39.4)	3 (9.1)	10 (30.3)	2 (6.1)
Pleural effusion	5 (15.2)	1 (3.0)	4 (12.1)	0
Atelectasis	3 (9.1)	0	3 (9.1)	0
Pulmonary infection	2 (6.0)	0	2 (6.0)	0
Intra-abdominal infection	3 (9.1)	0	2 (6.1)	1 (3.0)
Intra-abdominal fluid collections	2 (6.1)	1 (3.0)	1 (3.0)	0
Intra-abdominal bleeding	1 (3.0)	0	0	1 (3.0)
Anastomotic leakage	1 (3.0)	0	1 (3.0)	0
Seroma of the incision	1 (3.0)	1 (3.0)	0	0
Others[b]	3 (9.0)	0	3 (9.0)	0

[a] The two grade III surgical complications were both grade IIIb according to Clavien-Dindo classification. No grade IV or V surgical complications occurred.

[b] Others including acute cholecystitis, epidermal infection and acute attack of gout.

Supplementary Table 4. Relationship between PD-L1 or TMB at baseline and pathological response in the surgery set (N = 33).

	pCR	Non-pCR	P
CPS ≥1			>0.999
Yes (n = 9)	4 (44.4)	5 (55.6)	
No (n = 13)	5 (38.5)	8 (61.5)	
CPS ≥5			>0.999
Yes (n = 8)	3 (37.5)	5 (62.5)	
No (n = 14)	6 (42.9)	8 (57.1)	
CPS ≥10			0.648
Yes (n = 7)	2 (28.6)	5 (71.4)	
No (n = 15)	7 (46.7)	8 (53.3)	
TMB in total population[a]			0.021
≥median (n = 15)	9 (60.0)	6 (40.0)	
<median (n = 15)	2 (13.3)	13 (86.7)	
TMB in intestinal type[a]			0.021
≥median (n = 6)	6 (100)	0	
<median (n = 7)	2 (28.6)	5 (71.4)	
TMB in other types[a]			0.206
≥median (n = 9)	3 (33.3)	6 (66.7)	
<median (n = 8)	0	8 (100)	

Data are n (%). Two-sided Fisher Exact test was used to determine statistical significance between subgroups. PD-L1, programmed cell death-ligand 1; TMB, tumor mutation burden; CPS, combined positive score; pCR, pathological complete response.

[a] The median level of TMB was 4.04 mutation/Mb.

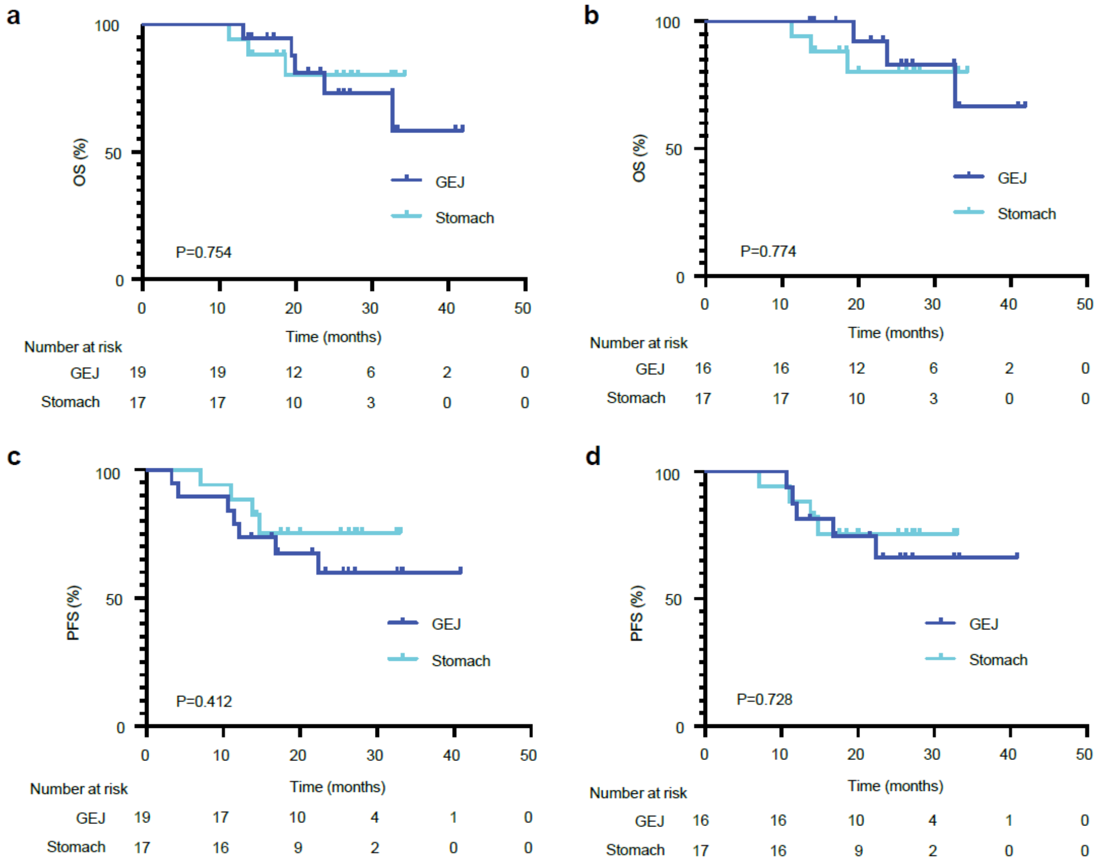

Supplementary Figure 1. Survival analysis in subgroup by tumor site. a OS in the full analysis set. Two-sided log-rank test was used to determine statistical significance between subgroups. **b** OS in the surgery set. Two-sided log-rank test was used to determine statistical significance between subgroups. **c** PFS in the full analysis set. Two-sided log-rank test was used to determine statistical significance between subgroups. **d** PFS in the surgery set. Two-sided log-rank test was used to determine statistical significance between subgroups. OS, overall survival; GEJ, gastroesophageal junction; PFS, progression-free survival. Source data are provided as a Source Data file.

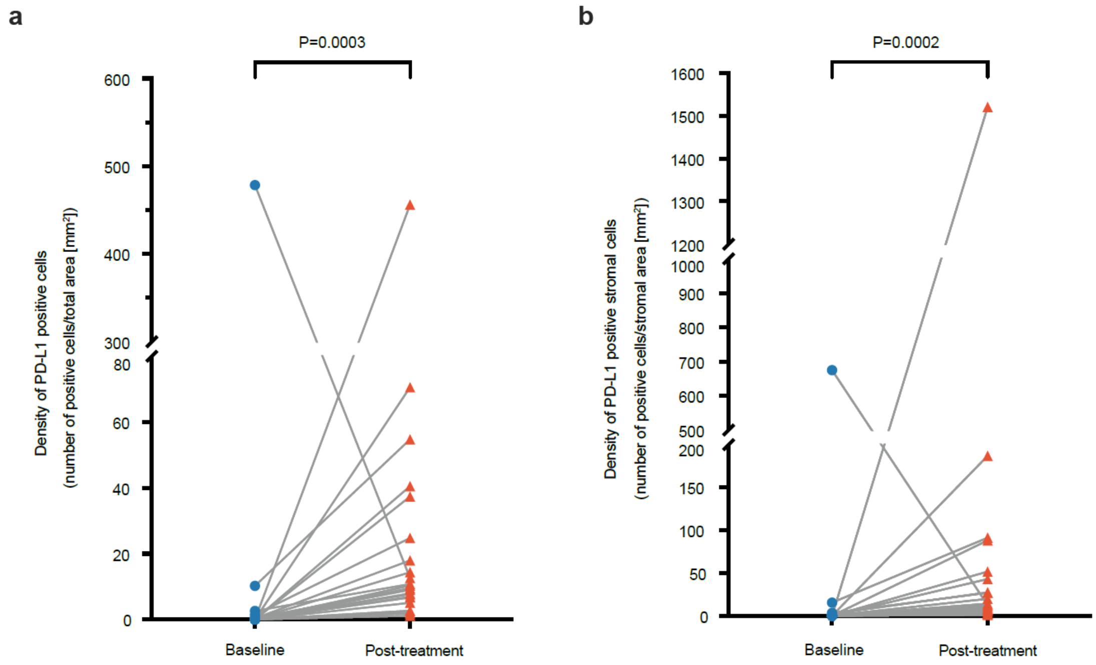

Supplementary Figure 2. Comparison of PD-L1 density before and after neoadjuvant therapy. a PD-L1 density in total area (n = 22 biologically independent samples). Calculated by two-sided paired t-test. Each line represents one patient. **b** PD-L1 density in stromal area (n = 22 biologically independent samples). Calculated by two-sided paired t-test. Each line represents one patient. PD-L1, programmed cell death-ligand 1. Source data are provided as a Source Data file.

A phase II study of neoadjuvant chemoradiotherapy plus anti-PD-1 antibody (SHR-1210) in locally advanced adenocarcinoma of stomach or gastroesophageal junction (Neo-PLANET)

Protocol number: SHR-1210-CRT-GC-IIT

Protocol version: 1.0

Protocol version date: May 14, 2018

Sponsor: Zhongshan Hospital, Fudan University

Study affiliation: Zhongshan Hospital, Fudan University

Principal investigators: Yihong Sun, Tianshu Liu

Table of contents

1. Background........**15**

1.1 The basis of the application of neoadjuvant chemoradiotherapy in locally advanced adenocarcinoma of stomach or gastroesophageal junction and the selection of specific regimens 15

1.1.1 The basis of the application of neoadjuvant chemoradiotherapy in locally advanced adenocarcinoma of stomach or gastroesophageal junction........15

1.1.2 Selection of the neoadjuvant chemotherapy regimen 15

1.1.3 Selection of the neoadjuvant concurrent chemoradiotherapy regimen 15

1.2 The basis of the application of SHR-1210 in locally advanced adenocarcinoma of stomach or gastroesophageal junction and dose selection........16

2. Study Objectives........**17**

2.1 Primary objective........17

2.2 Secondary objectives 17

2.3 Exploratory objectives 17

3. Study Protocol **17**

3.1 Overview of the study design 17

3.2 Study population........18

3.2.1 Inclusion criteria 18

3.2.2 Exclusion criteria 19

3.2.3 Withdrawal criteria 21

3.2.4 Criteria for early termination of the study by investigators 21

3.3 Sample size calculation........21

3.4 Study drugs, radiotherapy, and surgical treatment........22

3.4.1 Study drugs 22

3.4.1.1 Dose and administration time of the study drugs 22

3.4.1.2 Dose delay of the study drugs 22

3.4.1.3 Instructions for the use of the study drugs 22

3.4.1.4 Drug overdose or incorrect drug administration 23

3.4.2 Concurrent chemoradiotherapy........23

3.4.2.1 Dose and duration of concurrent chemoradiotherapy 23

3.4.2.2 Dose delay of radiotherapy........23

3.4.2.3 Dose adjustment of capecitabine........24

3.4.2.4 Radiotherapy technology........24

3.4.2.5 Target area setting 24

3.4.2.6 Radiation overdose or incorrect operation during radiotherapy........25

3.4.3 Surgical treatment 25

3.5 Study flow........25

3.5.1 Screening period 25

3.5.2 Treatment period........27

3.5.2.1 Baseline assessment 27

3.5.2.2 Evaluation during neoadjuvant therapy........27

3.5.2.3 Postoperative evaluation and treatment........28

3.5.3 Follow-up period after completion of study treatment....32
3.6 Study evaluation....32
3.6.1 Efficacy evaluation....32
3.6.2 Progression evaluation....33
3.6.3 Safety evaluation....33
3.6.3.1 Evaluating adverse events to chemoradiotherapy....33
3.6.3.2 Evaluating adverse events to surgery....34
3.7 Endpoints....34
3.7.1 Primary endpoint....34
3.7.2 Secondary endpoints....34
3.7.3 Exploratory endpoints....34
3.8 Concomitant medication and treatment....34
3.8.1 Symptomatic treatment and supportive treatment....34
3.8.2 Concomitant medication....35
4. Adverse Events....35
4.1 Adverse events and laboratory abnormalities....35
4.1.1 Adverse events....35
4.1.1.1 Drug-related adverse events....36
4.1.1.2 Radiotherapy-related adverse events....36
4.1.1.3 Surgery-related adverse events....36
4.1.2 Laboratory test abnormalities....36
4.1.3 Serious adverse events....36
4.2 Handling of safety indicators....37
4.2.1 Reporting of serious adverse events....37
4.2.2 Follow-up of adverse events....37
4.2.3 Follow-up of abnormal laboratory test values....37
4.2.4 Pregnancy....37
4.2.5 Disease progression....37
4.2.6 Death....37
4.3 Dose adjustment....37
4.3.1 Drug-related adverse events....37
4.3.1.1 Routine precautions for dose adjustment....38
4.3.1.2 Principle of dose adjustment for common adverse events....38
4.3.1.2.1 Principle of adjustment for drug-related specific adverse events....38
4.3.1.2.2 Dose adjustment for hematological toxicity....39
4.3.1.2.3 Dose adjustment for non-hematological toxicity....41
4.3.1.2.3.1 Routine precautions and dose adjustment for oxaliplatin toxicity 41
4.3.1.2.3.2 Routine precautions and dose adjustment for capecitabine toxicity 43
4.3.1.2.4 Routine precautions and dose adjustment for SHR-1210 toxicity....44
4.3.2 Radiotherapy-related adverse events....46
5. Statistical Analysis....46
5.1 Observation data and measurement data....46

5.2 Efficacy analysis 47
5.3 Safety analysis 47
5.4 Exploratory analysis 47

6. Study-related Ethics 47

6.1 Local regulations/Declaration of Helsinki 47
6.2 Review by the ethics committee 47
6.3 Informed consent 47

7. Drug and Specimen Management 48

7.1 Study drug SHR-1210 48
7.1.1 Storage 48
7.1.2 Inventory 48
7.2 Specimen management 48

8. Confidentiality Measures 48

9. References 48

1. Background

1.1 The basis of the application of neoadjuvant chemoradiotherapy in locally advanced adenocarcinoma of stomach or gastroesophageal junction and the selection of specific regimens

1.1.1 The basis of the application of neoadjuvant chemoradiotherapy in locally advanced adenocarcinoma of stomach or gastroesophageal junction

In recent years, a number of large-scale clinical studies have shown that neoadjuvant chemoradiotherapy has good efficacy and safety in the treatment of locally advanced adenocarcinoma of stomach or gastroesophageal junction (GEJ). A phase III clinical study (POET study) was conducted in Germany using preoperative chemoradiotherapy for the treatment of low esophageal and gastric cardia adenocarcinoma, and the results tend to indicate that preoperative chemoradiotherapy offers a survival advantage over preoperative chemotherapy. A number of multicenter clinical studies in the United States (such as RTOG-9904) have shown that preoperative chemoradiotherapy achieves good outcomes in locally advanced gastric cancer. Although the efficacy result have not yet been announced, the ongoing international multicenter TOPGEAR study has shown that preoperative neoadjuvant chemoradiotherapy (ECF-RT) does not significantly increase toxicity, treatment side effects, or surgical complications.

1.1.2 Selection of the neoadjuvant chemotherapy regimen

The neo-CLASSIC study (NCT01880632) conducted by our center in 2013 explored the effect of a capecitabine plus oxaliplatin (XELOX) regimen as neoadjuvant chemotherapy in treatment of locally advanced gastric cancer with regional lymph node metastasis. The preliminary results showed that the regimen achieved an objective tumor remission rate of 50%. Moreover, no serious adverse events (SAEs) occurred during the study (unpublished data). These results indicate that the XELOX neoadjuvant chemotherapy regimen is safe and effective in the treatment of locally advanced gastric cancer with regional lymph node metastasis. The subsequent follow-up will evaluate the long-term oncological effects of this regimen, and the results are anticipated. Based on the above studies, we plan to conduct neoadjuvant chemotherapy before and after concurrent chemoradiotherapy (induction chemotherapy and consolidation chemotherapy) using the XELOX regimen. The specific program is as follows: Capecitabine will be administered intermittently (chemotherapy for 2 weeks, drug withdrawal for 1 week) twice a day at 1000 mg/m^2. Oxaliplatin will be administered on the first day at a dose of 130 mg/m^2. The treatments will be repeated every 3 weeks.

1.1.3 Selection of the neoadjuvant concurrent chemoradiotherapy regimen

The SWOG9008/INT-0116 study was a phase III clinical randomized study conducted in Europe and America with landmark significance [1], making postoperative adjuvant chemoradiotherapy the standard adjuvant treatment for patients with T3, T4, and/or lymph node positive gastric cancer after D0/D1 resection. In this study, postoperative radiotherapy was performed concurrently with 5-fluorouracil (5-FU) treatment, and the dose of radiotherapy was 45 Gy/25 times/5 weeks. Fluorouracils are considered the standard drugs for sensitization of digestive tract tumors to concurrent radiotherapy and are recommended by the NCCN Guidelines for Gastric Cancer. Another study shows that 5-FU upregulates the expression of programmed death-ligand 1 (PD-L1) on the surface of gastrointestinal tumor cells [2]. At present, there is no high-level evidence to support that capecitabine, a novel oral fluorouracil drug, can be used alone for preoperative radiosensitization of gastric cancer. The application of capecitabine needs to be further verified by

clinical trials. In summary, this study intends to apply intensity-modulated radiotherapy to visible tumors and high-risk lymphatic drainage areas (a total dose of 45 Gy/25 times, 1.8 Gy/day). Oral capecitabine (850 mg/m^2, bid, po) will be administered concurrently for chemosensitization.

1.2 The basis of the application of SHR-1210 in locally advanced adenocarcinoma of stomach or gastroesophageal junction and dose selection

Anti-programmed cell death protein 1 (PD-1) immunotherapy has been proven to have clinical efficacy in treatment of a number of advanced cancers, including melanoma [3], non-small cell lung cancer (NSCLC) [4], and renal cell carcinoma (RCC) [5]. It has been approved in the United States, Europe, Japan, and other countries [6-9].

Preclinical data show that PD-1 targeted therapy improves antitumor activity, enhances the production level of interferon gamma (IFN-γ), and increases the proportion of CD4/CD8 tumor infiltrating T-effector cells that express PD-1 (Curran et al., 2010) [10]. Existing data from clinical trials of other anti-PD-1 monoclonal antibodies (such as pembrolizumab and nivolumab) show that the anti-PD-1 antibodies have controllable safety in patients with unresectable gastric/GEJ cancer and possess good antitumor activity. In addition, a number of clinical studies have shown that PD-1 antibodies/PD-L1 inhibitors have broad prospects in neoadjuvant treatment of cancers, such as triple negative breast cancer and non-small cell lung cancer.

SHR-1210 is a humanized anti-PD-1 antibody. It specifically blocks the binding of PD-1 and PD-L1 and terminates the PD-1 immunosuppressive signals induced by the interaction between PD-1 and PD-L1 in T cells. Cell experiments have shown that SHR-1210 effectively blocks the interaction of PD-1/PD-L1, promotes tuberculin-induced proliferation and activation of T cells in peripheral blood mononuclear cells (PBMC), and reverses the PD-1/PD-L1 mediated decrease in protein kinase B (AKT) phosphorylation. The antitumor activity of SHR-1210 has also been confirmed in a mouse model of MC38 colorectal cancer (the model is designed to express human PD-1) and a mouse xenograft model of U87-MG human malignant glioblastoma. Moreover, SHR-1210 was well-tolerated in these mice.

At present, 15 clinical studies of SHR-1210 are being carried out in people with advanced malignant tumors, including 1 study of combined radiotherapy and 3 studies of combined chemotherapy. The efficacy-related data are still being collected and analyzed, and the final results have not been released. To date, SHR-1210 has shown good safety in all subjects. The incidence of SAEs is only 1%. The incidence rates of SHR-1210-related adverse events (AEs), namely, immune-related SAE pneumonia and interstitial lung diseases, are 1.1% and 0.5% respectively.

SHR-1210-APA-01 is an ongoing phase I/II exploratory study designed to preliminarily evaluate the safety and efficacy of the combination of SHR-1210 and apatinib in subjects with advanced hepatocellular carcinoma (HCC) or gastric cancer (GC) that have failed standard antitumor treatment or when there is a lack of standard treatment. The dose selection of SHR-1210 in the regimen can be used as a reference. Namely, SHR-1210 is administered by intravenous infusion at 3 mg/kg or 200 mg once every 2 weeks (q2w). In addition, in this study, a combination of chemoradiotherapy and SHR-1210 will be used in the neoadjuvant therapy (the former itself may cause radiation pneumonitis, while the AEs related to the latter also include immune-related SAE pneumonia and interstitial lung diseases), which may cause the superposition of AEs. Moreover, the subjects will undergo subsequent surgery. Therefore, it is temporarily proposed that SHR-1210 will be administered via intravenous infusion at a dose of 200 mg, once every 3 weeks (q3w).

2. Study Objectives

2.1 Primary objective

Determination of the pathological complete response (pCR) rate achieved in patients with locally advanced (cT3-4aN+M0) adenocarcinoma of stomach or GEJ using the combination of SHR-1210 and chemoradiotherapy as a neoadjuvant therapy.

2.2 Secondary objectives

1) Determination of the total pathological complete response (tpCR) rate achieved using the combination of SHR-1210 and chemoradiotherapy as a neoadjuvant therapy.
2) Determination of the major pathological response (MPR) rate achieved using the combination of SHR-1210 and chemoradiotherapy as a neoadjuvant therapy.
3) Determination of the R0 resection rate achieved using the combination of SHR-1210 and chemoradiotherapy as a neoadjuvant therapy.
4) Determination of downstaging using the combination of SHR-1210 and chemoradiotherapy as a neoadjuvant therapy.
5) Determination of the progression-free survival (PFS)/overall survival (OS) achieved using the combination of SHR-1210 and chemoradiotherapy as a neoadjuvant therapy.
6) Determination of the safety of the combination of SHR-1210 and chemoradiotherapy as a neoadjuvant therapy.

2.3 Exploratory objectives

Exploration of the biomarker status of tumor tissues and blood samples obtained before and after SHR-1210 treatment, including but not limited to PD-L1, immune-related gene expression characteristics, and tumor mutation load, as well as their relationships with the disease status or the efficacy of the combination of SHR-1210 and chemoradiotherapy.

3. Study Protocol

3.1 Overview of the study design

This study is an open, single-arm, single-center, prospective phase II clinical study. It aims to evaluate the efficacy and safety of application of the neoadjuvant therapy (PD-1 antibody SHR-1210 plus chemoradiotherapy) followed by radical resection in patients with locally advanced (cT3-4aN+M0) adenocarcinoma of stomach or GEJ.

The selected patients will receive the following neoadjuvant chemoradiotherapy sequentially: (1) induction chemotherapy (3 w): 1 cycle of XELOX regimen (oxaliplatin 130 mg/m^2 d1+ capecitabine 1000 mg/m^2 bid*14 d, q21d); (2) concurrent chemoradiotherapy (5 w) within 1 week after the induction chemotherapy: intensity modulated radiotherapy will be applied to visible tumors and high-risk lymphatic drainage areas (total dose 45 Gy/25 times, 1.8 Gy/day), and capecitabine (850 mg/m^2, bid, po) will be administered orally as a chemosensitizer during this period; (3) consolidation chemotherapy 2-3 weeks after chemoradiotherapy: 1 cycle (3 w) of the XELOX regimen (oxaliplatin 130 mg/m^2 d1 + capecitabine 1000 mg/m^2 bid*14 d, q21d).

From d1 of induction chemotherapy to 3 weeks before surgery, the PD-1 antibody SHR-1210 (200 mg, iv, q3w) will be used in combination with chemoradiotherapy.

Re-evaluation will be performed 1-3 weeks after consolidation chemotherapy, and the resectable patients will receive D2 radical surgery.

Postoperative adjuvant treatment: We recommend 4 cycles of the XELOX regimen (oxaliplatin 130 mg/m^2 d1 + capecitabine 1000 mg/m^2 bid*14 d, q21d) 4-6 weeks after radical surgery.

Patients who develop local tumor progression and distant metastasis during treatment and thus become unable to undergo D2 radical surgery and patients who experience intolerable toxicity will be withdrawn from the study treatment. These patients will be given other appropriate treatments and subjected to follow-ups and evaluations. The PFS and OS of these withdrawn patients will be recorded. The enrolled patients will undergo tumor evaluation at baseline, after concurrent chemoradiotherapy, and before surgery. After radical surgery, the patients will be regularly followed up and evaluated for metastasis/recurrence and survival until death, the last date of known survival, or 3 years after the primary effectiveness analysis.

3.2 Study population

Patients who are initially diagnosed with locally advanced adenocarcinoma of stomach or GEJ.

3.2.1 Inclusion criteria

Patients included in this study must meet all of the following conditions:

1) Male or female, with an age of 18 to 75 years;
2) Having an Eastern Cooperative Oncology Group (ECOG) performance status of 0-1 within 7 days before enrollment;
3) Being histologically diagnosed with adenocarcinoma;
4) Having tumor lesions at stomach or GEJ (Siewert type II or III);
5) Being clinically staged as cT3-4aN+M0 (8th edition of the American Joint Commission on Cancer (AJCC) Tumor, Nodes, Metastasis (TNM) staging system) based on endoscopic ultrasonography or enhanced computed tomography (CT)/magnetic resonance imaging (MRI);
6) Having at least one evaluable lesion in abdominal CT/MRI examination according to Response Evaluation Criteria in Solid Tumors version 1.1 (RECIST v1.1) criteria;
7) The surgeons participating in this study decide that radical resection can be performed;
8) Physical condition and organ function allow the patients to undergo major abdominal surgery;
9) Routine blood and biochemical indices of the subjects examined within 7 days before enrollment meet the following standards:

   a. hemoglobin ≥90 g/L; absolute neutrophil count (ANC) $\geq 1.5\times10^9$/L; and platelet count $\geq 100\times10^9$/L (patients must not have received blood transfusion or growth factor support within 14 days before collecting the blood samples);

   b. alanine aminotransferase (ALT) and aspartate aminotransferase (AST) ≤2.5×upper limit of normal (ULN); and alkaline phosphatase (ALP) ≤ 2.5×ULN;

   c. serum total bilirubin <1.5×ULN (Patients with Gilbert syndrome can be enrolled if the total bilirubin is <3×ULN);

   d. serum creatinine <1.5×ULN or estimated glomerular filtration rate ≥60 mL/min/1.73 m^2;

   e. serum albumin ≥30 g/L; and

   f. international normalized ratio (INR) or prothrombin time (PT) ≤1.5×ULN, unless the patient is receiving anticoagulation therapy and the PT value is within the expected treatment range of anticoagulants; and

   g. Activated partial thromboplastin time (APTT) ≤1.5×ULN;
10) Having no serious concomitant diseases that render a survival time of <5 years;
11) Being willing and able to follow the protocol during the study;

12) Written informed consent must be provided before the subjects are enrolled in this study. Moreover, the patients understand that they can withdraw from the study at any time without any consequence;
13) Female patients with childbearing potential must have a negative urine or serum pregnancy test within 7 days before enrollment. Moreover, these female patients must agree to take efficient contraceptive measures during the study period, for at least 120 days after the last SHR-1210 administration and for at least 180 days after the last chemotherapy administration; and
14) Nonsterilized male patients must agree to take effective contraceptive measures during the study period, for at least 120 days after the last dose of SHR-1210 and for at least 180 days after the last dose of chemotherapy.

3.2.2 Exclusion criteria

Patients who meet any of the following conditions will be excluded:

1) Patients with signs of distant metastasis, or surgeons decide that radical resection cannot be performed (Bormann type 4 or large (≥8 cm) type 3; gastric cancer with extensive local lymph node metastasis [JGCA-bulky N2: at least one node of 3 cm or more in diameter, or at least three consecutive nodes each of diameter 1.5 cm or more, along the coeliac, splenic, common or proper hepatic arteries]; or gastric adenocarcinoma invading distal esophagus more than 3 cm);
2) Patients with T1 or T2 lesions indicated by imaging examination or ultrasonic gastroscopy;
3) Patients who have received cytotoxic chemotherapy, radiotherapy, immunotherapy, or radical surgery for the gastric adenocarcinoma, except corticosteroids;
4) Patients with active autoimmune disease or a history of refractory autoimmune disease.
   Note: Patients with the following diseases will not be excluded and may be subjected to further screening:
   a. controlled type 1 diabetes;
   b. hypothyroidism (if controllable by hormone replacement therapy alone);
   c. controlled celiac disease;
   d. skin diseases that do not require systemic treatment (e.g., vitiligo, psoriasis, hair loss); and
   e. any other disease that is not expected to recur in the absence of external triggering factors;
5) Patients with any active malignant tumors within 2 years, except the specific cancer being studied in this trial and locally recurrent cancers that have been cured (such as resected basal cell or squamous cell skin cancer, superficial bladder cancer, cervical, or breast carcinoma in situ);
6) Patients with uncontrollable pleural effusion, pericardial effusion, or ascites within 14 days before enrollment that require frequent drainage (the conditions are allowed to be determined by effusion cytology examination);
7) Patients who suffer digestive tract bleeding within 2 weeks before enrollment or who have been judged by the investigators to have high risk of bleeding;
8) Patients who have gastrointestinal perforation and/or fistula within 6 months before enrollment;
9) Patients who suffer upper gastrointestinal tract obstruction or functional abnormality or malabsorption syndrome that may affect the absorption of capecitabine;
10) Patients who lose ≥20% of body weight within 2 months before enrollment;
11) Patients with a history of the following lung diseases: interstitial pulmonary disease, noninfectious pneumonitis, pulmonary fibrosis, and acute pulmonary disease;

12) Patients with uncontrollable systemic diseases including diabetes and hypertension;
13) Patients who have severe chronic or active infections in need of systemic antibacterial, antifungal, or antiviral treatment, including tuberculosis and human immune deficiency virus (HIV) infection;
14) Patients who have untreated chronic hepatitis B or are chronic hepatitis B virus (HBV) carriers with HBV DNA greater than 500 IU/mL or are positive for hepatitis C virus (HCV) RNA shall be excluded. Patients who are carriers of inactive hepatitis B surface antigen (HBsAg), who have been treated and have stable hepatitis B (HBV DNA <500 IU/mL), and who are cured of hepatitis C can be enrolled;
15) Patients with any of the following cardiovascular risk factors should be excluded:
    a. occurrence of cardiogenic chest pain within 28 days before enrollment, which is defined as moderate pain sufficient to limit the instrumental activities of daily life;
    b. occurrence of a symptomatic pulmonary embolism within 28 days before enrollment;
    c. occurrence of acute myocardial infarction within 6 months before enrollment;
    d. having a history of New York Heart Association grade III/IV heart failure within 6 months before enrollment;
    e. occurrence of ventricular arrhythmia of grade 2 or higher within 6 months before enrollment or having concurrent supraventricular tachyarrhythmias in need of drug treatment; and
    f. occurrence of a cerebrovascular accident (CVA) within 6 months before enrollment;
16) Patients with grade ≥1 peripheral neuropathy according to the National Cancer Institute Common Terminology Criteria for Adverse Events (NCI-CTCAE) v4.03. However, patients who only show the disappearance of deep tendon reflex (DTR) do not have to be excluded;
17) Patients who suffer moderate or severe renal damage (creatinine clearance rate equal to or lower than 50 mL/min calculated according to the Cockcroft and Gault equation) or have serum creatinine >ULN;
18) Patients with dihydropyrimidine dehydrogenase (DPD) deficiency;
19) Patients who are allergic to any drug ingredients used in this study;
20) Patients who have received allogeneic stem cell transplantation or organ transplantation;
21) Patients who need systemic treatment with corticosteroids (prednisone at a dose higher than 10 mg/d or equivalent doses of similar drugs) or other immunosuppressants within 14 days before enrollment.
    Note: Patients who are currently using or have previously used any of the following steroid regimens can be enrolled:
    a. adrenaline replacement steroids (prednisone ≤10 mg/d or equivalent dose of similar drugs);
    b. local, ophthalmic, intra-articular, intranasal, and inhaled corticosteroids with minimal systemic absorption; and
    c. short-term (≤7 days) use of corticosteroids for prevention (e.g., prevention of allergy to contrast agents or antiemetics for specific chemotherapy) or for treatment of nonautoimmune diseases (e.g., delayed hypersensitivity reactions caused by contact allergens);
22) Patients who have received live vaccines within 4 weeks before enrollment (note: seasonal influenza vaccines are usually inactivated vaccines and are thus allowed; vaccines used in the nasal cavity are live vaccines and are not allowed);

23) Patients who have received immunotherapy (such as interleukin, interferon, thymine) or any trial treatment within 28 days or 5 half-lives (whichever is shorter, but no shorter than 14 days) before enrollment;
24) Patients who have received palliative radiotherapy within 14 days before enrollment;
25) Patients who have received anti-PD-1 treatment, anti-PD-L1 treatment, anti-PD-L2 treatment, or any other antibody or drug treatments that specifically target T cell costimulation or checkpoint pathway;
26) Patients who have received major surgery within 28 days before enrollment, unless the surgery is minimally invasive (e.g., peripherally inserted central catheter [PICC]);
27) For patients with uncontrolled epilepsy, central nervous system diseases or history of mental disorders, the investigators shall evaluate whether the clinical severity will impede the signing of informed consent or affect the patients' compliance with oral drugs; and
28) Patients with potential medical conditions or alcohol/drug abuse or dependence, which are considered by the investigators to hinder the administration of the study drug or affect the analysis of drug toxicity or the interpretation of AEs.

3.2.3 Withdrawal criteria

1) Subjects request to withdraw the informed consent.
2) The occurrence of local tumor progression and distant metastasis during the treatment period renders it impossible to perform D2 radical surgery.
3) Treatment is interrupted for more than 2 weeks due to toxic reaction to radiotherapy or interrupted for more than 6 weeks due to toxic reaction to chemotherapy.
4) Grade 2 toxicity occurs 4 times, grade 3 toxicity occurs 3 times, or grade 4 toxicity occurs during chemoradiotherapy.
5) The investigators or sponsors believe that the safety of patients may be endangered if she/he continues to receive the study treatment.
6) Patients are lost to follow-up or pregnancy occurs.
7) The subjects have poor compliance, such as simultaneous use of antitumor treatments other than the study treatment.

3.2.4 Criteria for early termination of the study by investigators

1) One treatment-related death occurs in the first stage of perioperative treatment;
2) Two treatment-related deaths occur during the overall perioperative treatment period;
3) During the first stage of preoperative treatment, more than 3 patients experience disease progression, or more than 2 patients are confirmed to have ultrarapid progression; or
4) During the overall preoperative treatment process, more than 6 patients experience disease progression, or more than 4 patients are confirmed to have ultrarapid progression.

Hyperprogression: Within 2 months after the start of the study treatment, 1) the tumor progresses; 2) the growth rate of the tumor is more than doubled; or 3) the volume of tumor increases > 50%.

3.3 Sample size calculation

This study adopted the Simon's minmax two-stage design, and the main study endpoint is the pCR rate. The null hypothesis is set to 16% [11], while the alternative hypothesis is set to 35%. Assuming the one-sided significance level of 0.05 and power of 83%, it is estimated that 15 subjects need to be recruited in the first stage. If ≥3 subjects achieve pCR, the study will enter the second stage and another 21 subjects will be added. The total sample size will be 36 patients. Overall, if 10 or more of the 36 subjects achieve pCR, further study deserves to be conducted.

3.4 Study drugs, radiotherapy, and surgical treatment

3.4.1 Study drugs

3.4.1.1 Dose and administration time of the study drugs

SHR-1210:

SHR-1210 will be administered by intravenous infusion at a dose of 200 mg per infusion. The first dose will be administered on day 1 of the induction chemotherapy. SHR-1210 will then be administered once every 3 weeks until 3 weeks before the radical operation. A total of 5 infusions will be given.

XELOX regime (one cycle before and after concurrent chemoradiotherapy)

Oxaliplatin: Oxaliplatin will be administered via a 3-h intravenous infusion at a dose of 130 mg/m^2. It will only be given on day 1 of the treatment course. Each treatment course will last 3 weeks.

Capecitabine: Capecitabine will be administered orally twice a day at a dose of 1000 mg/m^2 (equivalent to a total daily dose of 2000 mg/m^2). The first dose will be given in the evening on day 1 of the treatment course, and the last dose will be given in the morning on day 15. The drug will be administered intermittently. Each treatment course will last 3 weeks (oral administration for 2 weeks, drug withdrawal for 1 week).

(**Note:** The doses of capecitabine and oxaliplatin are calculated in milligrams based on the measured baseline body surface area (BSA). Although the weight of a patient may change slightly during the study period, the BSA will still be calculated according to the baseline measurement. Unless a patient's weight change is considered an adverse drug reaction or exceeds 10% of the baseline value, the doses will not be adjusted based on the weight change.)

3.4.1.2 Dose delay of the study drugs

In case of a delay in SHR-1210 administration:

1. If an SHR-1210 administration is delayed ≤ 10 d, the subsequent administrations shall be carried out according to the original medication plan;
2. If an SHR-1210 administration is delayed >10 d, the timing of the subsequent administrations shall be rescheduled according to the single administration time.

Note: The time interval between the last SHR-1210 administration and the surgery shall not be less than 2 weeks.

If relevant AEs occur during the treatment with capecitabine and oxaliplatin, the administration of the drugs shall be delayed until the following occur:

1) neutrophil count ≥1500/mm^3 and platelet count ≥ 75,000/mm^3;

2) any nonhematological toxicity (except hair loss) returns to the baseline level or grade ≤1; or

3) in the case of prolonged toxicity, patients will be withdrawn from the study due to toxic response if the administration of all chemotherapy drugs needs to be delayed or interrupted for more than 6 weeks during the treatment period. If only oxaliplatin needs to be stopped, the original study plan will be carried out.

3.4.1.3 Instructions for the use of the study drugs

Oxaliplatin

1) Oxaliplatin administration shall be completed via a 2-h intravenous infusion on day 1 of the treatment course before the first dose of capecitabine is administered.

2) The investigators must calculate the dose based on the patient's BSA. The dose of oxaliplatin shall be as close as possible to the dose calculated at 130 mg per square meter of BSA.

3) Oxaliplatin administration does not require prehydration. In the case of accidental overdose, the

administration must be stopped immediately.

Capecitabine

1) The investigators need to first calculate a patient's BSA according to his/her actual height and weight and then select the appropriate dose according to the capecitabine dose table.
2) Capecitabine shall be taken orally within 30 min after meals (breakfast, dinner).
3) Capecitabine shall be taken with 150-200 mL of water (not with juice).

SHR-1210

1) SHR-1210 is a white or whitish lyophilized powder with a dose of 200 mg/bottle. It is packaged in 20-mL USP type I glass vials, and the concentration of active drug ingredient is 40 mg/mL. Each vial of preparation will be reconstituted with 5.0 mL of sterile water for injection, and the concentration after dissolution will be 40 mg/mL. The reconstituted preparation will be further diluted with an intravenous injection mix containing 5% glucose to a final concentration of 0.5-10 mg/mL. The diluted preparation will be packaged in infusion bags.
2) After the first 2 infusions, the patients must be observed for at least 1 h in a place equipped with resuscitation equipment and emergency drugs. Starting from the third infusion, the patients must be observed for at least 30 min in areas equipped with resuscitation equipment and rescue drugs.
3) The duration of SHR-1210 infusion shall be controlled to be within 20-60 min, preferably 30 min.
4) SHR-1210 shall not be administered simultaneously with any other drug. If a patient needs to undergo other drug treatments or radiotherapy on the same day, SHR-1210 shall be administered first.

3.4.1.4 Drug overdose or incorrect drug administration

Administration of the drugs in quantities greater than the limit dose or incorrect operation during drug administration shall be recorded in the patients' record sheets and appropriate electronic case report form (eCRF). AEs related to the drug overdose or incorrect operation during drug administration will be recorded in the AE eCRF. If the occurrence of drug overdose or incorrect operation is accompanied by an AE with significant safety impact, the sponsors or designated personnel must be notified within 24 h of discovery through the SAE reporting procedure. Appropriate supportive treatments shall be given according to the conditions.

3.4.2 Concurrent chemoradiotherapy

3.4.2.1 Dose and duration of concurrent chemoradiotherapy

Intensity modulated radiation therapy will be used. The total dose will be 45 Gy and the single dose will be 1.8 Gy. The radiation therapy will last for 5 weeks (once a day from Monday to Friday, a total of 25 treatments). During concurrent chemoradiotherapy, capecitabine will be administered each day in which radiotherapy treatments are given. Capecitabine will be administered orally twice a day in the morning and evening at a dose of 850 mg/m^2 (equivalent to a total daily dose of 1700 mg/m^2).

3.4.2.2 Dose delay of radiotherapy

1) The treatment may be temporarily interrupted upon the occurrence of severe treatment toxicity. When the toxicity is relieved, the patients shall receive the planned dose. It must be recorded if a patient cannot receive the planned dose due to severe toxic reactions.
2) Temporary interruption of treatment shall be allowed when due to therapeutic toxicity. The treatment may be temporarily interrupted when leukocyte count is lower than 2.0×10^9/L, platelet count is lower than 50×10^9/L with a tendency to bleed, vomiting occurs more than 3 times/day after antiemetic treatment, or significant weight loss occurs (a 10% decrease compared to the body weight

before treatment).

3) Radiotherapy shall be continued once the patients recover from the toxic reaction. A patient will be withdrawn from the study if the interval between radiotherapy treatments is greater than 2 weeks.

3.4.2.3 Dose adjustment of capecitabine

Principles for dose adjustment of capecitabine for hematologic adverse events

It is generally believed that capecitabine will not aggravate neutropenia/agranulocytosis or excessively extend their duration. Therefore, capecitabine will continue to be administered if less than grade 2 hematologic toxicity is noted in an unplanned laboratory test during the chemotherapy cycle. In addition, capecitabine shall be discontinued if grade 3 or 4 hematologic toxicity events occurs during the chemotherapy cycle. The next chemotherapy cycle can be started only when the hematologic toxicity has returned to grade <2. In the case of anemia (nonhemolytic), there shall be no need to reduce the dose or interrupt the treatment because the condition can be managed satisfactorily by blood transfusion.

Principles for dose adjustment of capecitabine for non-hematologic adverse events

Capecitabine shall be discontinued immediately if a grade 2, 3, or 4 non-hematologic AE occurs (unless the investigators have determined that such AE is only related to oxaliplatin), and further measures shall be taken according to the instructions below. Chemotherapy shall be stopped if the creatinine clearance rate drops to <30 mL/min during the treatment. Once reduced, the dose of capecitabine shall not be added back unless oxaliplatin is permanently discontinued. (**Note:** The duration when capecitabine chemotherapy is discontinued is regarded as lost treatment days. Afterward, chemotherapy shall be carried out according to the predetermined chemotherapy schedule. The doses missed during the interruption of chemotherapy will not be compensated.)

3.4.2.4 Radiotherapy technology

1) The design of the radiation field employs the intensity-modulated radiation therapy technology. Through CT simulation, the images are transmitted to the treatment planning system (TPS) to outline the target area to be irradiated, namely, the clinical target volume (CTV). The doctors determine the prescribed dosage and the restricted dosage for vital organs, and based on which, the physicists make the reverse intensity modulation plan through the TPS. The doctors then select the relatively optimal plan to treat patients.

2) Before radiotherapy, patients will be instructed to increase breathing frequency to reduce respiratory intensity and decrease the movement of internal abdominal organs during radiotherapy.

3) Localization shall be consistent with the treatment time, preferably fasting or 3-4 h after a meal.

4) Patients will be instructed to adopt a supine position and will be immobilized by a vacuum cushion with hands wrapping around the elbows on the forehead. A laser light will be positioned.

5) The scanning range will be from the level of the sternum angle to the level of the umbilicus, and the scanning layer interval will be 3 mm.

6) Multifield concentric radiotherapy will be performed using high-energy X-rays (≥6 MV).

7) The dose-volume histogram (DVH) will be used to evaluate the conformity of the target areas and the dose received by normal tissues and organs.

3.4.2.5 Target area setting

1) The gross tumor volume (GTV) includes the primary lesions and the visible metastatic lymph nodes, which will be delineated using CT/MRI in combination with gastroscopy.

2) CTV includes GTV and high-risk lymph node areas.

a. Target area must include the adjacent pancreatic body and at least 3 cm of the distal esophagus.

b. The range of the high-risk lymph node areas is designated in accordance with the NCCN gastric cancer treatment guidelines (V5. 2017), including the adjacent paraesophageal and perigastric lymph nodes (1-6, and 7 groups), suprapancreatic lymph nodes (12 groups), celiac trunk lymph nodes (9 groups), and splenic hilar lymph nodes (10 groups).

3) The planning target volume (PTV) is an expansion of the CTV:

a. in the left and right directions (X): CTV + 0.5 cm;

b. in the cranial and caudal directions (Y): CTV + 1.0 cm; and

c. in the thoracic and abdominal directions (z): CTV + 0.5 cm.

3.4.2.6 Radiation overdose or incorrect operation during radiotherapy

Any over-the-limit radiotherapy dose (which is defined as a total dose of the following: spinal cord D_{max} ≥45 Gy; kidney $D_{33\%}$ >15-20 Gy; liver D_{mean} >23 Gy, and V_{20} >50%) or incorrect operation during radiotherapy shall be recorded in the patients' record sheets and the appropriate eCRF. AEs related to radiotherapy overdose or incorrect operation during radiotherapy will be recorded in the AE eCRF. If the occurrence of radiotherapy overdose or incorrect operation leads to an AE with significant safety impact, the sponsors or designated personnel must be notified within 24 h of discovery through the SAE reporting procedure. Appropriate supportive treatment shall be given according to the conditions.

3.4.3 Surgical treatment

Radical treatment will aim at tumor R0 resection, and the surgical method will be ~~total~~ gastrectomy + D2 lymph node dissection (at least 25 lymph nodes will be removed, and the lymph nodes that are removed in groups during the operation will be collected and 22reserved). The method of digestive tract reconstruction is not specified. The reconstruction shall be performed in accordance with the clinical diagnosis and treatment standards. For patients undergoing radical treatment, appropriate surgical incisions shall be selected for good exposure. Careful intraoperative exploration, accurate intraoperative staging, and abdominal exfoliative cytology will be carried out. The postoperative pathology report shall include the efficacy of preoperative chemotherapy.

3.5 Study flow

3.5.1 Screening period

1) The subjects will be informed, sign an informed consent form, and be assigned a selection number.

2) Tumor evaluation shall be performed within 2 weeks before enrollment using appropriate repeatable methods, such as CT/MRI, gastroscopy, and pathological evaluation.

3) The following assessments shall also be performed within 2 weeks before enrollment: demographic data, gastric cancer/treatment history, concomitant diseases/treatments, full physical examination (including vital signs, ECOG performance status, height, weight, and systemic nervous system examination), laboratory tests (blood routine/biochemistry, fecal routine + occult blood test, urine routine, coagulation function, tumor markers, thyroid function, hepatitis markers), electrocardiogram (ECG), heart ultrasound, and a pregnancy test (for all women not in menopause or in menopause less than 12 months).

4) Lung function tests will be performed on patients who are suspected or known to have severe respiratory diseases or patients who exhibit significant respiratory symptoms unrelated to the underlying cancer, including but not limited to spirometry tests and assessment of pulmonary diffusion volume in the screening period. These tests will help to determine whether a patient is suitable to participate in this study.

5) After completion of screening all items, the investigators must review the results/data before enrolling the subjects (see the table below).

**Screening items**	**-14**	**Duration of screening (no more than 14 days)**
**Demographic data**	●	
**Medical history**		
Diagnosis and treatment history of gastric cancer	●	
Concomitant diseases and treatments	●	
**Full physical examination**	●	Including vital signs, ECOG performance status, height, weight, and systemic nervous system examination
**Laboratory examination**		
Urine pregnancy test	●	Urine pregnancy test will be performed in women of childbearing age
Urine routine	●	
Blood routine/biochemistry	●	
Coagulation function	●	
Tumor markers	●	
Fecal routine + occult blood	●	
Thyroid function	●	
Hepatitis markers	●	
**ECG**	●	
**Heart ultrasound**	●	
**Lung function test (optional)**	●	For patients who are suspected or known to have severe respiratory diseases or patients who exhibit significant respiratory symptoms unrelated to the underlying cancer
**Tumor diagnosis**		
Gastroscopy	●	Tumor location and size shall be determined by gastroscopy
Ultrasonic gastroscopy (optional)	●	Ultrasonic gastroscopy must be performed when the depth of tumor invasion cannot be determined by abdominal MRI and CT
Pathology	●	Including Lauren classification

Tumor staging		
Abdominal and pelvic CT	●	Female patients need to undergo enhanced pelvic CT
Chest CT	●	
Tumor evaluation	●	Contrast-enhanced CT/MRI of the abdomen is a mandatory examination, and there must be assessable lesions according to the RECIST v1.1.
**After completion of screening all the items, the investigators must review the results/data before enrolling the subjects.**		

3.5.2 Treatment period

3.5.2.1 Baseline assessment

1) The baseline examination shall be performed within 7 days after the patients sign the informed consent form and are enrolled in the study. Chemotherapy must be performed within 7 days after enrollment.

2) The following assessments shall be completed within 1 week before treatment: vital signs (temperature, blood pressure, and heart rate), physical examination (including ECOG performance status, height, weight, and systemic nervous system examination), blood routine/biochemical examination (including the calculation of creatinine clearance rate), coagulation function, fecal routine and occult blood, urine routine and pregnancy test, tumor markers, thyroid function, hepatitis markers, ECG, heart ultrasonography, and lung function (optional).

3) According to the above schedule, the existing laboratory tests performed in the screening period and the auxiliary examinations performed within 7 days before chemotherapy can be used as the baseline without the need for repeated examinations.

4) Tumor tissues must be collected and archived for subsequent pathological remission assessment, as well as the analysis of PD-L1 and other biomarkers. If archived samples are not available, fresh tumor biopsy specimens must be collected at baseline. In addition, 5 mL of whole blood must be collected in the baseline period. Tumor tissue samples and blood samples will be processed and submitted for examination in accordance with the requirements of the central laboratory.

5) The breast CT, abdominal and pelvic MRI/CT, and gastroscopy performed during the screening period will be used as the baseline for tumor assessment.

3.5.2.2 Evaluation during neoadjuvant therapy

1) AEs will be continuously monitored during the study treatment period until 28 days after the last study treatment.

2) During the study treatment period, the patients' concomitant diseases and treatments and their compliance with the study treatment will be continuously monitored.

3) The following data will be collected from each patient at specified times: vital signs, weight, Karnofsky score, hematological (including leukocytes/neutrophils) and serum biochemical examinations, coagulation function, thyroid function, fecal routine and occult blood test, urine routine, tumor marker examination, and hepatitis markers.

4) ECG, heart ultrasonography, chest X-ray and pulmonary function tests will be performed when there are clinical indications.

5) All patients will be evaluated for tumor treatment efficacy (abdominal and pelvic CT/MRI + chest

CT) at specified times. The efficacy will be judged according to the RECIST v1.1. Patients who have progressed will be withdrawn from the study, whereas patients who have not progressed will continue to undergo the neoadjuvant treatment or surgery.

6) During the study treatment period and within 28 days after the last study treatment, information including hospitalization status and duration, treatment of the AEs, and the situation and time of outpatient treatment will be recorded.

3.5.2.3 Postoperative evaluation and treatment

1) After the patients undergo radical resection, the tumor tissues will be collected and hematoxylin and eosin (HE)-stained sections will be prepared. Pathological experts will compare these sections with the HE-stained tumor tissue sections collected at baseline and evaluate the pathological remission rate.

2) It will be regarded as disease progression if a patient is confirmed postoperatively to have metastatic gastric cancer (such as the detection of tumor cells in ascites exfoliative cytology) or if the effect to achieve R0 resection fails for a patient. In these cases, the intraoperative findings, surgical methods, and postoperative pathology shall be recorded in detail regardless of what surgical method is used.

3) Timely evaluation of tumors is recommended after surgery (including physical examination, tumor markers, chest CT, and abdominal and pelvic CT/MRI). If a patient has signs of recurrence (such as relevant clinical manifestations), additional tumor evaluation must be performed during the treatment period. Any possible reoperations or/and further cancer treatments must also be recorded.

4) Postoperative adjuvant treatment: We recommend starting a 4-cycle XELOX regimen (oxaliplatin 130 mg/m^2 d1+ capecitabine 1000 mg/m^2 bid*14 d, q21d) 4-6 weeks after radical surgery.

5) In the case that radical resection of gastric cancer (D2 lymph node dissection) cannot be performed due to tumor progression, we recommend switching to paclitaxel-based systemic chemotherapy or conduct treatment according to the investigators’ clinical practice. The disease-free survival (DFS) and overall survival (OS) shall be recorded. If R0 resection is not achieved, we recommend postoperative supplementary radiotherapy or treatment according to the investigators' clinical practice. Patients who have not undergone radical gastric cancer treatment (D2 lymph node dissection) due to other reasons will be treated following the clinical practice of the research center. Again, the DFS and OS shall be recorded (see the table below).

**Course of treatment**	**-1 w**	**1 w**	**2 w**	**3 w**	**4 w**	**5 w**	**6 w**	**7 w**	**8 w**	**9 w**	**10 w**	**11 w**	**12 w**	**13 w**	**14 w**	**15 w**	**16 w**
	**Baseline (c)**	**Preoperative treatment (g)**															**Surgery**
		**SHR-1210**			**SHR-1210**			**SHR-1210**			**SHR-1210**			**SHR-1210**			
		**XELOX**			**Concurrent chemoradiotherapy**					**Evaluation**		**XELOX**				**Evaluation**	
Informed consent (a)	●																
Full physical examination (b)	●				●	●	●	●	●	●		●				●	
Laboratory tests (d)																	
Urine pregnancy test	●	In the presence of clinical indication															
Blood routine/biochemistry	●				●	●	●	●	●	●		●				●	
Tumor Marker (h)	●									●						●	
Coagulation function	●				●		●		●	●		●				●	
Urine routine	●				●		●		●	●		●				●	
Fecal routine + occult blood	●				●		●		●	●		●				●	

Thyroid function	●	In the presence of clinical indication								●	In the presence of clinical indication					●	
Hepatitis markers	●	In the presence of clinical indication								●	In the presence of clinical indication					●	
ECG	●	In the presence of clinical indication														●	
Heart ultrasound	●	In the presence of clinical indication														●	
Lung function (optional)	In the presence of clinical indication																
Tumor evaluation (e) (f)																	
Abdominal and pelvic CT/MRI	●									●						●	
Chest CT	●									●						●	
Blood sample collection	●																
Specimen pathological evaluation	●																●
AE and treatment	Recording is required during the treatment period and within 28 d after study treatment ends																

(a) Informed consent must be obtained before any research procedures may begin. Chemotherapy shall be performed within 7 d after enrollment.

(b) Full physical examination includes vital signs, ECOG performance status, height, weight, and systemic nervous system examination.

(c) The items screened within 7 d before enrollment can be used as baseline items, and there is no need to repeat the examination.

(d) Investigators must review the hematology and blood biochemistry results before surgery and before the start of each chemotherapy cycle (the blood test window is 3 days).

(e) X-ray, MRI, or CT scans shall be performed within 2 weeks before enrollment in each center (the baseline). The first efficacy evaluation shall be performed in the 9th week after the start of treatment (window period +/- 7 days). The efficacy will be judged according to the RECIST v1.1. Patients who have progressed will be withdrawn from the study, whereas patients who do not progress will be continued with neoadjuvant therapy or surgery.

(f) In the 15th week after the start of treatment, the second efficacy evaluation shall be performed, and the feasibility of surgery shall be determined. Radical resection shall be performed within 3 weeks (+/-7 days) after capecitabine is discontinued in the last cycle of chemotherapy before the evaluation. Postoperative chemotherapy will not be specified, but the XELOX regimen is recommended.

(g) If the preoperative treatment is delayed or suspended due to AEs or other reasons, the possibility of surgical resection shall be evaluated after drug withdrawal. If the tumor cannot be resected, the study will be terminated, and the observation period shall begin.

(h) The tumor markers examined include carcinoembryonic antigen (CEA), carbohydrate antigen 19-9 (CA19-9), and alpha-fetoprotein (AFP). The same tumor markers will be examined in subsequent tests.

3.5.3 Follow-up period after completion of study treatment

1) Follow-ups shall be conducted at the specified times (which are recommended to include physical examination, tumor marker examination, chest CT, and abdominal and pelvic CT/MRI). If a patient has signs of recurrence (such as relevant clinical manifestations), additional tumor evaluations must be performed during the treatment period. Any possible reoperations or/and further cancer treatments must be recorded.

2) No other cytotoxic agents shall be administered during the relapse-free follow-up period.

3) The examinations can be carried out within +/- 4 weeks of the specified dates.

4) The study follow-up period will end after 3 years. Afterward, follow-up will be conducted every 6 months. After 5 years, follow-up will be conducted once a year. The recurrence and survival of patients will be followed up until the death of the patients, the last known date of survival, or 2 years after the primary effectiveness analysis (see the table below).

**Follow-up evaluation**					
Time after enrollment (d)	**0.5 year**	**1 year**	**1.5 years**	**2 years**	**≥3 years (e)**
Tumor evaluation (a) (b)	●	●	●	●	●
Survival	●	●	●	●	●
Other cancer treatments (c)	Any occurrence shall be recorded.				

(a) If the patient has signs of recurrence (such as clinical symptoms), additional tumor evaluation is required. All evaluations used to diagnose tumor recurrence/new tumors shall be recorded in the case record form, including tumor evaluations not specified by the protocol.

(b) During the follow-up period, evaluations will be carried out based on the clinical reality. It is recommended to include CT or MRI

(c) No other cytotoxic agents are allowed during the study treatment period and during the relapse-free follow-up period.

(d) Examinations can be carried out ± 4 weeks from the specified dates.

(e) The study follow-up period will end after 3 years. Afterward, follow-up will be conducted every 6 months. After 5 years, follow-up will be conducted once a year. The recurrence and survival of patients will be followed up until the death of the patients, the last known date of survival, or 2 years after the primary effectiveness analysis.

3.6 Study evaluation

3.6.1 Efficacy evaluation

As one of the evaluation methods, baseline and postoperative specimens will be pathologically evaluated in this study. The pathological response will be graded according to the Becker’s tumor regression grade system: grade 1a, no residual tumor/tumor bed; grade 1b, <10% re residual

tumor/tumor bed; grade 2, 10%-50% residual tumor/tumor bed; grade 3 >50% residual tumor/tumor bed.

1) pCR is defined as the proportion of patients with no residual tumor (grade 1a) at primary tumor.

2) tpCR is defined as the proportion of patients with no residual tumor (grade 1a) at both primary tumor and lymph nodes.

3) MPR is defined as the proportion of patients with <10% residual tumor at primary tumor (including grade 1a and 1b).

4) R0 resection rate was defined as the proportion of patients whose resected specimen showed negative margin.

5) Downstaging: The proportion of patients with pathological downstaging.

Another method of efficacy evaluation is based on the results of abdominal and pelvic CT/MRI and chest CT at various time points. The consistency of CT/MRI examinations must be ensured when evaluating each patient, and the same examination techniques shall be used during the study. During the study, each patient shall be evaluated by the same investigator according to RECIST v1.1. The survival of the enrolled patients will be included in the efficacy evaluation, and the specific definitions are as follows:

6) PFS is defined as the time from the initiation of neoadjuvant therapy to disease recurrence, progression, or any-cause death; and

7) OS is defined as the time from the initiation of neoadjuvant therapy to any-cause death.

3.6.2 Progression evaluation

The occurrence of any of the following conditions can be regarded as disease progression or recurrence:

1) Evidence shows that patient's primary tumor lesion has reappeared;

2) Evidence shows that a patient has developed new gastric cancer;

3) Evidence shows that a patient has developed distant metastasis;

4) All-cause death occurs; and

5) There is tumor progression according to the RECIST v1.1.

The occurrence of any of the following conditions is defined as tumor hyperprogression:

1) Tumor progression occurs within 2 months after start of the study treatment;

2) Tumor growth rate increases more than 2-fold within 2 months after start of the study treatment; and

3) Tumor volume increases more than 50% within 2 months after start of the study treatment.

Note: tumor progression or recurrence should be confirmed by cytological or histological examination if possible. Without the support of other objective indicators (i.e., radiological, histological/cytological examination), the elevation of tumor markers alone or unexplained clinical deterioration cannot be regarded as evidence for recurrence. The date of recurrence is defined as the date on which the objective examinations yield an affirmative result.

3.6.3 Safety Evaluation

3.6.3.1 Evaluating adverse events to chemoradiotherapy

Throughout the study, AEs related to chemoradiotherapy will be graded and recorded according to NCI-CTCAE v4.03. The description of toxicity characteristics will include the severity, duration, and time of occurrence. The main AEs include the following: vomiting, diarrhea, anemia, leukopenia, thrombocytopenia, hand-foot syndrome, cutaneous capillary hyperplasia, immune-related AEs (including pneumonia, interstitial lung disease, elevated AST, elevated ALT, skin rash,

diarrhea, hypothyroidism, and hyperthyroidism), and hyperprogression of tumors. SAEs refer to the occurrence of the following adverse medical conditions under any dose: 1) death; 2) life threatening conditions; 3) hospitalization or an extended hospital stay; 4) deformity/disability; 5) congenital abnormalities or birth defects; or 6) medical events considered important by investigators based on medical judgment.

3.6.3.2 Evaluating adverse events to surgery

Surgery-related AEs include intraoperative and postoperative complications (referring to surgery-related complications that occur within 30 days after surgery. If the hospital stay exceeds 30 days, the observation time will be extended until discharge). Among the postoperative complications, only serious complications will be observed, including abdominal or gastrointestinal tract bleeding, anastomotic fistula, grade B and greater pancreatic fistula, and incision complications (infection, bleeding, and dehiscence). The severity is graded according to the Clavien–Dindo classification.

3.7 Endpoints

3.7.1 Primary endpoint

- pCR is defined as the proportion of patients with no residual tumor (grade 1a) at primary tumor.

3.7.2 Secondary endpoints

- tpCR is defined as the proportion of patients with no residual tumor (grade 1a) at both primary tumor and lymph nodes.
- MPR is defined as the proportion of patients with <10% residual tumor at primary tumor (including grade 1a and 1b).
- R0 resection rate was defined as the proportion of patients whose resected specimen showed negative margin.
- Downstaging: The proportion of patients with pathological downstaging.
- PFS is defined as the time interval from the date of patient enrollment to the date when tumor progression or censoring is recorded for the first time.
- OS is defined as the time interval from patient enrollment to all-cause death or censoring.
- The incidence and severity of AEs is evaluated according to NCI-CTCAE v4.03.

3.7.3 Exploratory endpoints

- The status of biomarkers in SHR-1210 tumor tissues and blood samples, including but not limited to PD-L1, the expression characteristics of immune-related genes and tumor mutation load, as well as their relationships with the disease status or the efficacy of the combination of SHR-1210 and chemoradiotherapy, will be explored.

3.8 Concomitant medication and treatment

All treatments necessary for the health and safety of the patients can be determined by the investigators according to local medical standards. In addition, any diagnosis, treatment, or surgical operation during the study period shall be recorded, including the date, indications, operating instructions, and all clinical findings.

3.8.1 Symptomatic treatment and supportive treatment

Any disease-related symptoms developed by the patients during this study will receive the necessary palliative and supportive treatments, including the administration of epoetin and other hematopoietic growth factors (such as colony stimulating factors [CSF]), blood transfusions and blood transfusion products, antibiotics, antiemetics, and other applicable drugs. The treatments will be carried out according to local standard treatment guidelines or procedures.

3.8.2 Concomitant medication

During the screening and study periods, the administration of the following drugs shall be prohibited or restricted:

- **Immunosuppressants** (except those used to treat drug-related AEs);
- **Systemic corticosteroids at more than 10 mg/day** (except those used for the treatment or control of drug-related AEs or those used in the short term as preventive treatment (such as antiemetic treatment for specific chemotherapy));
- **Live vaccines**: Live vaccines are prohibited from 28 days before the first administration of the study drug to 60 days after the last administration;
- **Drugs with ototoxicity or nephrotoxicity**: Concomitant drugs that may have ototoxic or nephrotoxic effects (such as aminoglycosides, cephaloridine, furosemide, and amphotericin B) shall be avoided or properly monitored;
- **Allopurinol**: The interaction between 5-FU and allopurinol has been observed and may reduce the efficacy of 5-FU;
- **Metronidazole**: Study has shown that metronidazole increases the toxicity of 5-FU in patients with colorectal cancer through reducing the clearance rate of antitumor drugs (Bardakji, 1986). It shall be used with caution in the cancer patients participating in this study;
- **Coumarin derivative anticoagulants**: Changes in coagulation parameters and/or bleeding have been reported in patients receiving capecitabine and concomitant coumarin derivative anticoagulants (such as warfarin and phenprocoumon). Coumarin derivative anticoagulants shall be used with caution in the cancer patients participating in this study;
- **Antiviral and antiprotozoal drugs**: Capecitabine shall not be used concurrently with the antiviral drug sorivudine or its chemical analogues (such as brivudine). It has been reported in the literature that sorivudine inhibits the activity of dihydropyrimidine dehydrogenase (DPD). Such interaction increases the toxicity of fluoropyrimidine and may be lethal. Metronidazole increases the toxicity of fluorouracil in patients with colorectal cancer, which the investigators shall pay attention to during the trial;
- **Digestive tract drugs**: A study has shown that, after 4 weeks of preadministration of cimetidine, oral or intravenous administration of fluorouracil leads to an increase in its plasma concentration. This phenomenon may be the combined effect of liver enzyme inhibition and hepatic blood flow reduction. Caution shall be exercised upon the combined administration of these 2 drugs.
- **Other drugs that affect liver and kidney functions**: Other drugs (including alcohol) that affect liver and kidney functions shall be administered with caution during the use of the study drugs. In addition, liver and kidney function shall be closely monitored during the medication period;
- **Other anticancer treatments**: During the chemotherapy period of the study (if a patient does not withdraw from the study due to tumor recurrence) or the disease-free follow-up period, treatment of gastric cancer with other cytotoxic drugs, study drugs, or radiotherapy is prohibited;
- **Herbs with immunostimulatory properties (such as mistletoe extract)**; and
- **Herbs known to affect the function of vital organs (such as hypericin)**

4. Adverse Events

4.1 Adverse events and laboratory abnormalities

4.1.1 Adverse events

AEs refer to any unexpected medical events that occur in patients or clinical research cases after receiving a certain treatment that do not necessarily have a causal relationship with the

treatment. Therefore, AEs can be any unpleasant and/or unanticipated signs (including abnormal laboratory test results, see section 4.1.2), symptoms, or diseases temporally related to the treatment, regardless of whether they are related to the medication. The deterioration of the existing disease during the study period shall also be reported as an adverse event.

All clinical AEs encountered during the clinical study shall be recorded on the AE pages of the eCRF. The severity of the AEs (except surgery-related AEs) will be graded according to NCI-CTCAE v4.03 and recorded in the eCRF in detail. If an AE is not included in the NCI-CTCAE, it will be graded using the following four-point scoring system (mild, moderate, severe, and critical).

Mild	Discomfort but no impact on normal daily activities
Moderate	Discomfort sufficient to reduce or affect daily activities
Severe	Inability to work or carry out normal daily activities
Critical	Immediate life-threatening
Death	Adverse event-related death

In addition, the relationship between an adverse event and treatment shall be evaluated.

4.1.1.1 Drug-related adverse events

The main AEs include the following: vomiting, diarrhea, anemia, leukopenia, thrombocytopenia, hand-foot syndrome, cutaneous capillary hyperplasia, immune-related AEs (including pneumonia, interstitial lung disease, elevated AST, elevated ALT, skin rash, diarrhea, hypothyroidism, and hyperthyroidism), and tumor hyperprogression (using the following scale: 1, tumor progresses; 2, tumor growth rate is increased more than 2-fold; or 3, tumor volume is enlarged by more than 50% within 2 months after start of the study treatment).

4.1.1.2 Radiotherapy-related adverse events

Radiotherapy-related AEs include the following: gastrointestinal tract toxicity (nausea, vomiting, decreased appetite, esophageal mucositis, and diarrhea); hematological toxicity (leucopenia, anemia, and thrombocytopenia); and abnormal liver function.

4.1.1.3 Surgery-related adverse events

Surgery-related AEs include intraoperative and postoperative complications (referring to surgery-related complications that occur within 30 days after surgery. If a hospital stay exceeds 30 days, the observation time will be extended until discharge). Among the postoperative complications, only serious complications will be observed, including abdominal or gastrointestinal tract bleeding, anastomotic fistula, grade B and greater pancreatic fistula, and incision complications (infection, bleeding, and dehiscence). The severity is graded according to the Clavien–Dindo classification.

4.1.2 Laboratory test abnormalities

The laboratory test results will be recorded on the Laboratory Test Result page of the eCRF. In the following cases, abnormal laboratory test results will be recorded on the Adverse events page of the eCRF as adverse events: the appearance of clinical symptoms that require treatment or a change in the existing treatment; the need to discontinue/postpone, adjust the dose, or permanently discontinue the study chemotherapy drug; or the occurrence of SAEs.

4.1.3 Serious adverse events

SAEs refer to the following adverse medical conditions occurring under any dose: conditions that lead to death; conditions that is life threatening; conditions that require hospitalization or extended hospitalization; conditions that cause deformity/disability; conditions that cause congenital abnormalities or birth defects; and medical events that are deemed important by the investigators based on medical judgment.

4.2 Handling of safety indicators

4.2.1 Reporting of serious adverse events

Any SAEs or abnormal laboratory test results that occur during the study treatment period or within 28 days after the last administration of the drugs must be reported to the relevant department within 24 h of discovery, regardless of whether it is related to the treatment received by the patients.

4.2.2 Follow-up of adverse events

Even if the study treatment of a subject has been completed, all AEs (and the treatments) must be recorded entirely during the study treatment period and within 28 days after the last drug administration and must be followed up until the symptoms are completely relieved or fully interpreted. Irrelevant, mild, or moderate AEs must be followed up until 28 days after the last administration of the study drugs. Severe, critical, or related events must be followed up until the remission or stabilization of the events, patient death, initiation of new cancer treatments, or reassessment of the correlation.

4.2.3 Follow-up of abnormal laboratory test values

In the case of unexplainable abnormal laboratory test values, the test shall be repeated and followed up until the value returns to the normal range or baseline level, and/or a reasonable explanation of the abnormal test value is found. Once identified, the definite explanation shall also be recorded on the eCRF.

4.2.4 Pregnancy

It must be explained to female patients that if they become pregnant during the study, they must discontinue all trial drugs and report to the investigators immediately. In addition, the investigators must be informed if pregnancy occurs within 90 days after completion of the administration of trial drugs. The investigators shall report to the sponsors within 24 h. The investigators shall inform the patients of the risk of continuing pregnancy and the possible impact on the fetus. The investigators shall follow up with the patients until the termination of pregnancy. Pregnant patients shall be withdrawn from the treatment stage of the study and enter the follow-up period. Pregnancy of the spouse of a patient participating in the study shall also be reported to the investigators and the sponsors. The spouse shall be notified of the information above and be followed up.

4.2.5 Disease progression

A patient shall be withdrawn from the study when there is confirmed disease progression. The patient will receive further evaluation and treatment according to the existing guidelines and the clinical practice of the investigators, and the process shall be recorded. The AEs related to the disease progression shall be reported according to AE terminology. For example, if a patient develops epilepsy that is confirmed to be related to brain metastases, the term "seizures" shall be recorded as the AE rather than disease progression or brain metastasis. Deaths that are deemed by the investigators to be possibly caused by disease progression shall be recorded in the eCRF.

4.2.6 Death

In the case that death is recorded as an SAE, the AEs that involve symptomatic deterioration and the AEs that cause or promote fatal outcomes shall be recorded in the eCRF. If the cause of death is unknown and cannot be determined at the time of reporting, it shall be recorded as "death of unknown cause.”

4.3 Dose adjustment

4.3.1 Drug-related adverse events

4.3.1.1 Routine precautions for dose adjustment

The expected toxicity and dose reduction tables have been described in the study scheme. Toxicity will be graded according to the NCI-CTCAE v4.03.

Treatment delay or dose adjustment (after adverse events) are described below. The reasons for dose adjustment or delay, the supportive treatment measures adopted, and the results shall be recorded on the patient record card and eCRF.

General precautions

1) For AEs that manifested at baseline, the dose can be adjusted according to the change in toxicity grade if the investigators deem it necessary. For example, if a patient has a baseline fatigue level of 1 and experience a fatigue level of 2 during treatment, the dose shall be adjusted according to grade 1 toxicity.

2) For toxicity (such as hair loss or appetite change) that the investigators deem to be unlikely to develop into severe or critical events, the treatment will continue with the original dose (no dose reduction or interruption). In addition, no dose reduction or interruption is necessary in the case of anemia (nonhemolytic), because the condition can be satisfactorily improved by blood transfusion.

3) Upon the simultaneous occurrence of toxicities of different grades or severity, dose adjustment shall be carried out using the maximum reducible dose.

4) If the administration of all study drugs has to be delayed or interrupted for more than 6 weeks during the drug treatment period, the patient will be withdrawn from the study due to toxic reaction. The study protocol can still be carried out if only one drug is discontinued. However, protocols involving the discontinuation of capecitabine are not allowed.

5) When a patient has a non-hematological AE with a severity of grade 3 or greater and it is uncertain whether to carry out treatment with the original dose or with a reduced dose, discussion with the sponsors is require before continuing the treatment.

6) There may be a situation during treatment that the drug administration must be suspended because the patient has not recovered from drug toxicity. The duration of each suspension and the cumulative suspension time in each drug administration cycle shall not exceed 1 week, while the frequency of drug suspension in each administration cycle shall not exceed 2 times. The purpose is to ensure the drug intensity received by the subjects during treatment (the trial shall be terminated if the above criteria are exceeded, including the patients whose treatment is delayed for more than 2 weeks in the next cycle).

7) If the investigators have determined that a certain toxicity is caused by one drug (e.g., neurotoxicity is attributed to oxaliplatin), the doses of other drugs do not need to be adjusted.

8) When dose adjustment is needed due to a single abnormal hematology laboratory test value, the dose will be adjusted according to the hematological indices at the beginning of the treatment cycle. Since there is no plan to follow up blood routine during the chemotherapy cycles, the lowest values will not be collected.

9) At the beginning of a chemotherapy cycle, if the treatment has to be delayed due to either oxaliplatin or capecitabine, both the treatment with oxaliplatin and the treatment with capecitabine shall be delayed (except the oxaliplatin-related neurological AEs). Treatment can be resumed only when the conditions for restarting both oxaliplatin and capecitabine are met or when oxaliplatin must be stopped while the conditions for restarting capecitabine are met.

4.3.1.2 Principle of dose adjustment for common adverse events

4.3.1.2.1 Principle of adjustment for drug-related specific adverse events

1) In the case of cutaneous capillary hyperplasia (grade 2 with bleeding or grade 3) and immune-related AEs, SHR-1210 administration shall be suspended first when appropriate. SHR-1210

administration will be resumed once the toxicity returns to ≤ grade 1 or baseline level (specifically for the increase in laboratory test indicators such as ALT/AST and total bilirubin [TBIL]).

2) If the following events occur during the trial, SHR-1210 must be discontinued permanently: grade ≥3 immune pneumonia, grade ≥3 TBIL increase, grade 4 ALT/AST increase and other grade 4 immune-related AEs, or grade 4 infusion reactions. If SHR-1210 administration has been suspended for more than 12 weeks due to immune-related toxicity and the toxicity is still not reduced to grade ≤1 or baseline level, SHR-1210 must be discontinued permanently.
3) During the trial, if a subject develops fever (>38 °C) in need of corrective medication or has obvious symptoms of asthma, shortness of breath, or suffocation, the current or next scheduled SHR-1210 administration shall be delayed until the subject recovers from the symptoms. After the symptoms are relieved and stabilized for more than 7 days, SHR-1210 administration may be resumed. If necessary, pneumonia shall be excluded through imaging examination before SHR-1210 administration.
4) If neurotoxicity occurs, it is preferred to reduce the dose of oxaliplatin.

4.3.1.2.2 Dose adjustment for hematological toxicity

The doses of capecitabine and oxaliplatin shall be adjusted simultaneously according to the guidelines in Tables 1, 2, and 3.

It is generally believed that capecitabine does not aggravate neutropenia/agranulocytosis or excessively prolong its duration. Therefore, if a hematological toxicity of grade <2 is detected in unplanned laboratory tests during the chemotherapy cycles, capecitabine administration will continue. In addition, if grade 3 or 4 hematological toxicity occurs during the chemotherapy cycles, capecitabine shall be discontinued. The next chemotherapy cycle can be started only when the hematological toxicity has returned to <2. In the case of anemia (non-hemolytic), there shall be no need to reduce the dose or interrupt the treatment because the condition can be managed satisfactorily through blood transfusion.

Table 1 Dose adjustment for the occurrence of febrile neutropenia during chemotherapy

	**Grade 3**<br>**ANC <1.0×10^9/L accompanied by body temperature ≥ 38.5 °C**	**Grade 4**<br>**ANC <1.0×10^9/L, accompanied by a fever ≥ 38.5 °C**<br>**and life-threatening sepsis**
First occurrence	capecitabine at 75% of the original dose +<br>oxaliplatin 85 mg/m^2	Permanent cessation of treatment, unless the best treatment option for the patient is 50% of the original dose of capecitabine + 85 mg/m^2 oxaliplatin.
Second occurrence	Permanent cessation of treatment, unless the best treatment option for the patient is 50% of the original dose of capecitabine + 85 mg/m^2 oxaliplatin	Permanent cessation of treatment
Chemotherapy can be started only after the toxicity (except anemia) is relieved to grade ≤1 (ANC >1.5 ×10^9/L, platelet count >75×10^9/L).		

Table 2 Dose adjustment for the occurrence of neutropenia during treatment

	**Grade 2**<br>**1.0 ≤ANC <1.5×10^9/L**	**Grade 3**<br>**0.5 ≤ANC <1.0×10^9/L**	**Grade 4**<br>**ANC <0.5×10^9/L**
**<u>Laboratory test values before the start of the chemotherapy cycle</u>**: Chemotherapy shall be postponed until ANC is ≥1.5×10^9/L, platelets count is ≥75×10^9/L, and nonhematological toxicity returns to baseline or grade ≤1. Chemotherapy shall then be started at the following doses:			
First occurrence	No dose adjustment	capecitabine at 75% of the original dose + oxaliplatin 100 mg/m^2	capecitabine at 50% of the original dose + oxaliplatin 85 mg/m^2
Second occurrence	No dose adjustment	capecitabine at 75% of the original dose + oxaliplatin 85 mg/m^2	Permanent cessation of treatment
Third occurrence	No dose adjustment	Permanent cessation of treatment, unless the investigators decide after consideration in the best interest of the patient that the best treatment option for the patient is single drug chemotherapy with 75% of the original dose of capecitabine.	N/A

Table 3 Dose adjustment for the occurrence of thrombocytopenia and anemia during treatment

**Thrombocytopenia**	**Platelet count ≥50 - <75×10^9/L**	**Platelet count ≥25 - <50×10^9/L**	**Platelet count <25×10^9/L**
**<u>Laboratory test values before the start of the chemotherapy cycle</u>**: Chemotherapy shall be postponed until ANC is ≥1.5×10^9/L, platelets count is ≥75×10^9/L, and nonhematological toxicity returns to baseline or grade ≤1. Chemotherapy shall then be started at the following doses:			
First occurrence	Adjustment of oxaliplatin dose to 100 mg/m^2, with unchanged dose of capecitabine.	Capecitabine at 75% of the original dose + oxaliplatin 100 mg/m^2	Capecitabine at 50% of the original dose + oxaliplatin 85 mg/m^2
Second occurrence	Adjustment of oxaliplatin dose to 100 mg/m^2, with	Capecitabine at 75% of the original dose + oxaliplatin 85 mg/m^2	Permanent cessation of treatment, unless the investigators decide to continue chemotherapy

	unchanged dose of capecitabine.		after consideration of the best interest of the patient.<br>50% of the original dose of capecitabine
**Anemia (nonhemolytic) at any time during treatment**	**Grade 2 Hemoglobin 8.0 - <10.0 g/dL**	**Grade 3 Hemoglobin 6.5 - <8.0 g/dL**	**Grade 4 Hemoglobin <6.5 g/dL**
Occurrence at anytime	No dose adjustment (condition can be corrected through blood transfusion)	No dose adjustment (condition can be corrected through blood transfusion)	No dose adjustment (condition can be corrected through blood transfusion)

4.3.1.2.3 Dose adjustment for non-hematological toxicity

4.3.1.2.3.1 Routine precautions and dose adjustment for oxaliplatin toxicity

Peripheral neuropathy

Oxaliplatin is often accompanied by two types of peripheral neuropathy, including paresthesias and hypoesthesias of the hands and feet and around the mouth. Patients treated with oxaliplatin chemotherapy shall be informed to avoid cold drinks and exposure to cold water and air, especially within 3-5 days after oxaliplatin administration. For peripheral neuropathy, dose adjustment will be determined according to the scores in Table 4.

Table 4 Scoring the neurotoxicity of oxaliplatin

**Toxicity**	**Grade**	**Duration of toxicity**		
		**1 - 7 days**	**>7 days**	**Persistent during chemotherapy cycles**[a]
Paresthesia/hypoesthesia[b], no impact on function	1	No dose reduction	No dose reduction	No dose reduction
Paresthesia/hypoesthesia[b], affecting function but not the activities of daily living (ADL)	2	No dose reduction	No dose reduction	100 mg/m^2
Paresthesia/hypoesthesia[b] accompanied by pain, affecting function and ADL	3	No dose reduction	100 mg/m^2	Permanent cessation of treatment
Disabling or life-threatening persistent paresthesia/hypoesthesia	4	Permanent cessation of treatment	Permanent cessation of treatment	Permanent cessation of treatment

Toxicity	Grade	Duration of toxicity		
		1 - 7 days	>7 days	Persistent during chemotherapy cycles[a]
Acute throat paresthesia (during or after the 2-h infusion)[b]		Extension of the duration of next infusion to 6 h	N/A	N/A
a Toxicity is not relieved before the start of the next cycle.<br>b Paresthesia/hypoesthesia may be caused by cold.<br>c The condition can be pretreated with benzodiazepines.				

Throat paresthesia

The neurotoxicity of oxaliplatin can be manifested as a special type of throat paresthesia. There is no objective evidence of respiratory distress (hypoxia, laryngospasm, or bronchospasm) only the subjective symptoms of shortness of breath (acute respiratory distress). Such neurotoxicity can be induced or exacerbated by cold.

If a patient experience throat paresthesia, the blood oxygen saturation level shall be measured with a transcutaneous oximeter. If the reading is normal, this reading must be confirmed one additional time. Patients who are confirmed twice to have a normal blood oxygen saturation level may be given benzodiazepines or other antianxiety drugs. Patients shall be followed up until the symptoms are completely relieved. Oxaliplatin can then continue to be infused at 1/3 of the original rate. Because this syndrome may be related to the over-rapid infusion of oxaliplatin, the duration of oxaliplatin infusion shall be extended to 6 h in the subsequent cycle (instead of the usual 2-h infusion).

Patients receiving oxaliplatin shall not eat cold drinks on the first day of each cycle, because oxaliplatin may induce perioral or laryngeal paresthesia and acute pharyngeal paresthesia.

Other non-hematological adverse events related to oxaliplatin

Table 5 Dose adjustment of oxaliplatin for non-hematological adverse events

Toxicity	Grade	Dose adjustment
* Allergic reaction	Any	Permanent cessation of treatment
* Respiratory symptoms suggestive of pulmonary fibrosis	Any	Discontinuation of treatment and identification of the cause of the symptoms
* Interstitial pulmonary fibrosis not present at baseline	Any	Permanent cessation of treatment
Nausea and/or vomiting	4	100 mg/m^2

Toxicity	Grade	Dose adjustment
Diarrhea	3 or 4	100 mg/m^2
Stomatitis	4	100 mg/m^2
* No dose adjustment of capecitabine is necessary (for the best interest of the patient).		

Specific treatment delay related to oxaliplatin

If there is only one toxicity (paresthesia with pain or persistent dysfunction, see Table 8) at the beginning of a course of treatment, oxaliplatin chemotherapy shall be postponed and capecitabine monochemotherapy shall be continued. If the neurotoxicity persists until the next scheduled chemotherapy cycle, oxaliplatin shall be permanently discontinued. Investigators may consider continuing the capecitabine monochemotherapy.

Increase in capecitabine dose after discontinuation of oxaliplatin

If capecitabine monochemotherapy is continued after the discontinuation of oxaliplatin and there is a lack of tumor progression, capecitabine monochemotherapy shall be continued under the same dose for at least 1 cycle. Thereafter, if no grade 2, 3, or 4 toxicity occurs, the previously reduced capecitabine dose can be added back once a cycle in a step-by-step manner (from 50% to 75% of the baseline dose and then from 75% to 100% of the baseline dose). The added dose of capecitabine shall not exceed 100% of the baseline dose.

4.3.1.2.3.2 Routine precautions and dose adjustment for capecitabine toxicity

In the case of grade 2, 3, or 4 non-hematological AE, capecitabine shall be discontinued immediately (unless the investigators have determined that this toxicity is only related to oxaliplatin). Further measures shall then be taken according to the instructions below.

If the creatinine clearance rate drops to <30 mL/min during chemotherapy, the chemotherapy shall be stopped.

Once the dose of capecitabine is reduced, capecitabine shall not be increased again unless oxaliplatin is discontinued permanently.

(**Note:** The duration when capecitabine chemotherapy is discontinued is regarded as lost treatment days. Afterward, chemotherapy shall be carried out according to the predetermined chemotherapy schedule. The doses missed during the interruption of chemotherapy will not be compensated.)

Table 6 Dose adjustment of capecitabine for non-hematological adverse events

	Grade 2	Grade 3	Grade 4
First occurrence	No dose reduction; application of preventive measure if possible	Capecitabine administration at 75% of the original dose; application of preventive measure if possible	Permanent cessation of treatment unless the investigators decide to continue chemotherapy after consideration of the best interest of the patient. In this case, capecitabine shall be

	Grade 2	Grade 3	Grade 4
			administered at 50% of the original dose.
Second occurrence	Capecitabine administration at 75% of the original dose	Capecitabine administration at 50% of the original dose	
Third occurrence	Capecitabine administration at 50% of the original dose	Permanent cessation of treatment unless the investigators decide to continue chemotherapy after consideration of the best interest of the patient.	
Fourth occurrence	Permanent cessation of treatment unless the investigators decide to continue chemotherapy based on the consideration of the best interest of the patient.		

Note: Chemotherapy must be discontinued if toxicity of grade ≥2 occurs. Chemotherapy cannot be resumed until the toxicity returns to grade ≤1.

4.3.1.2.4 Routine precautions and dose adjustment for SHR-1210 toxicity

Cutaneous capillary hyperplasia

Please refer to "3.8.1 Symptomatic treatment and supportive treatment" for details.

Immune-related adverse events

Pneumonia	
Grade 2 pneumonia	Temporary suspension of SHR-1210 administration, which may be resumed when the condition recovers to grade 0-1
Grade 3 or 4 pneumonia	Permanent cessation of SHR-1210 administration
Enteritis	
Grade 2 diarrhea or colitis	Temporary suspension of SHR-1210 administration, which may be resumed when the condition recovers to grade 0-1
Grade 3 diarrhea or colitis	Temporary suspension of SHR-1210 administration, which may be resumed when the condition recovers to grade 0-1
Grade 3 diarrhea or colitis	Permanent cessation of SHR-1210

	administration
Hepatitis	
Patients without HCC or liver metastasis:<br>3×ULN < ALT/AST ≤5×ULN,<br>or 1.5×ULN < total bilirubin ≤3×ULN<br>Patients with HCC or liver metastasis:<br>•If the baseline ALT/AST is within the normal range, which then rises to 3×ULN < ALT/AST ≤5×ULN;<br>• If the baseline ALT/AST is 1×ULN < ALT/AST ≤3×ULN, which then rises to 5×ULN <ALT/AST ≤10×ULN<br>• If the baseline ALT/AST is 3×ULN < ALT/AST ≤5×ULN, which then rises to 8×ULN < ALT/AST ≤10×ULN	Temporary suspension of SHR-1210 administration, which may be resumed when the condition returns to the baseline level
Patients without HCC or liver metastasis:<br>ALT/AST >5×ULN or total bilirubin >3×ULN<br>Patients with HCC or liver metastasis:<br>ALT/AST >10×ULN or total bilirubin >3×ULN	Permanent cessation of SHR-1210 administration
Hypophysitis	
Grade 2 or 3 hypophysitis	Temporary suspension of SHR-1210 administration, which may be resumed when the condition recovers to grade 0-1
Grade 4 hypophysitis	Permanent cessation of SHR-1210 administration
Adrenal insufficiency	
Grade 2 adrenal insufficiency	Temporary suspension of SHR-1210 administration, which may be resumed when the condition recovers to grade 0-1
Grade 3 and 4 adrenal insufficiency	Permanent cessation of SHR-1210 administration
Type 1 diabetes mellitus	
Grade 3 hyperglycemia	Temporary suspension of SHR-1210 administration, which may be resumed when the condition recovers to grade 0-1
Grade 4 hyperglycemia	Permanent cessation of SHR-1210 administration
Skin	
Grade 3 rash or suspected Stevens–Johnson syndrome (SJS) or toxic epidermal necrolysis (TEN)	Temporary suspension of SHR-1210 administration, which may be resumed when the condition recovers to grade 0-1
Grade 4 rash or confirmed SJS or TEN	Permanent cessation of SHR-1210 administration

Encephalitis	
Newly developed moderate to severe neurological symptoms or signs	Temporary suspension of SHR-1210 administration, which may be resumed when the condition recovers to grade 0-1
Immune-mediated encephalitis	Permanent cessation of SHR-1210 administration
Others	
Grade 3 adverse event that occurs for the first time	Temporary suspension of SHR-1210 administration, which may be resumed when the condition recovers to grade 0-1
Recurrence of the same grade 3 adverse event	Permanent cessation of SHR-1210 administration
Life-threatening or grade 4 adverse events	Permanent cessation of SHR-1210 administration
Grade 3 myocarditis	Permanent cessation of SHR-1210 administration
Adverse events that require treatment with at least 10 mg of prednisone or its equivalent for more than 12 weeks	Permanent cessation of SHR-1210 administration
Grade 2 or 3 adverse events that last 12 weeks or longer	Permanent cessation of SHR-1210 administration
Due to drug-related toxicity, a maximum of 2 suspensions of administration are allowed. If adverse events that require suspension of drug administration occur again,	Permanent cessation of SHR-1210 administration

Tumor hyperprogression

Patients who experience tumor hyperprogression during the treatment period will be withdrawn from the study treatment. They will receive other appropriate treatments according to the clinical center's practice, and be followed up and evaluated. Their PFS and OS will be recorded.

4.3.2 Radiotherapy-related adverse events

Please refer to "3.4.2.2 Dose delay of radiotherapy" for details.

5. Statistical Analysis

A p value of less than 0.05 indicates that the difference tested is statistically significant. The confidence interval is 95%.

5.1 Observation data and measurement data

The observation data and measurement data obtained at the baseline and in each evaluation will include age, sex, height, weight, disease stage, ECOG performance status, lymph node status, and laboratory test indicators. The continuous variables in these data will be described using the mean, standard deviation, median, P25, P75, and minimum and maximum values, while the categorical variables will be described using frequency and relative frequency. A 95% confidence interval will be calculated for certain important indicators.

5.2 Efficacy analysis

This study will use the Kaplan–Meier method to estimate the 3-year PFS/OS rate and construct

the Kaplan-Meier curve. In addition, this study will estimate the median PFS/OS and the 95% confidence interval.

5.3 Safety analysis

When analyzing the safety of the combination of SHR-1210 and chemoradiotherapy as a neoadjuvant treatment, this study will mainly use descriptive statistical analysis, including describing and estimating the following: the incidence of various AEs (such as treatment-related death) and AEs (such as vomiting and leucopenia); the incidence of AEs of varying severity (grade 1-4); the relationship between the occurrence of AEs and the treatments performed at various stages of the study; and the vital signs and laboratory test indicators that are normal before treatments but abnormal after treatments as well as their relationship with the treatments when abnormal changes occur.

5.4 Exploratory analysis

This study intends to describe and compare the efficacy of the combination of SHR-1210 and chemoradiotherapy between patients with different tumor tissues and blood biomarker statuses (including but not limited to PD-L1, immune-related gene expression characteristics, and tumor mutation burden).

6. Study-related Ethics

6.1 Local regulations/Declaration of Helsinki

The investigators will ensure that the implementation of this study is in full compliance with the principles of the Declaration of Helsinki or the laws of the country where the institute is located, no matter what provisions the country has for the protection of human rights. This study will strictly follow the "Guideline for Good Clinical Practice" ICH Tripartite Guideline (January 1997) or local laws, whichever is more stringent.

6.2 Review by the ethics committee

The protocol, written informed consent form, and data directly related to the subjects must be submitted to the ethics committee, and the study can only be carried out after obtaining written approval from the ethics committee. The investigators must submit study report to the ethics committee at least annually (if applicable). Upon suspension and/or completion of the study, the investigators must notify the ethics committee in writing. The investigators must report all changes occurring during the study (such as the revision of the protocol and/or informed consent form) to the ethics committee in a timely manner and shall not implement these changes without the approval of the ethics committee, unless the changes are made to eliminate obvious and direct risks to the subjects. In such cases, the ethics committee shall be notified.

6.3 Informed consent

The investigators must provide the subjects or their legal representatives with an easy-to-understand informed consent form approved by the ethics committee and give the subjects or their legal representatives sufficient time to consider this study. A subject shall not be enrolled until a signed written informed consent is obtained from the subject. All updated versions of the informed consent form and written information will be provided to the subjects during their participation. The informed consent form shall be kept as an important document of the clinical trial for future reference.

7 Drug and specimen management

7.1 Study drug SHR-1210

7.1.1 Storage

The lyophilized preparation of SHR-1210 shall be stored at 2-8 °C (36-46 °F) and away from light. The reconstituted preparation will be stable for 24 h when stored at 2-8 °C (36-46 °F) in the dark. The diluted preparation (concentration range 0.5-10 mg/mL) can be stable for up to 24 h when stored in the infusion bag at 2-8 °C (36-46 °F). However, the diluted preparation will only stay stable for no more than 3 h during infusion at room temperature.

7.1.2 Inventory

SHR-1210 will be provided by the sponsor/collaborator Jiangsu HengRui Medicine Co., Ltd. The research center will confirm the receipt of SHR-1210 through interactive response technology (IRT) and verify the shipping conditions and contents. Any drugs damaged or lost during transportation will be replaced. SHR-1210 will be processed in the research center in accordance with the standard operating procedures of the center or returned to the sponsor with appropriate records. The method of destruction of SHR-1210 used by the research center must be approved by the sponsor. The research center must obtain the written authorization from the sponsor before SHR-1210 is destroyed, and the destruction of SHR-1210 must be recorded on the corresponding form. The SHR-1210 received, distributed, returned, and disposed shall be accurately recorded in the drug inventory log of the research center.

7.2 Specimen management

The fresh tissue samples of the patients will be prepared into formalin-fixed paraffin-embedded (FFPE) tissue blocks according to the standard operating procedures of the research center. FFPE sections will be prepared and stained as needed. The patients' baseline pathological specimens and blood samples as well as surgical pathological samples will be uniformly numbered and properly stored in the central laboratory of our hospital. These samples will eventually be tested in qualified local laboratories.

8. Confidentiality Measures

The results of this study may be published in medical journals, but we will keep the patients' information confidential in accordance with legal requirements. Unless required by relevant laws, the patients' personal information will not be disclosed. When necessary, the government management department, the hospital ethics committee, and the relevant personnel may assess patient information according to regulations.

9. References

[1] Macdonald JS, Smalley SR, Benedetti J, et al. Chemoradiation after surgery compared with surgery alone for adenocarcinoma of the stomach or gastroesophageal junction. N Engl J Med 2001;345:725–30

[2] Lauren Van Der Kraak, Gaurav Goel, Krishnaveni Ramanan, et al. 5-Fluorouracil upregulates cell surface B7-H1 (PD-L1) expression in gastrointestinal cancers. Journal for ImmunoTherapy of Cancer 2016; 4:65.

[3] Weber JS, D'Angelo SP, Minor D, Hodi FS, et al. Nivolumab versus chemotherapy in patients with advanced melanoma who progressed after anti-CTLA-4 treatment (CheckMate 037): a randomised, controlled, open-label, phase 3 trial. Lancet Oncol 2015 Apr;16(4):375-84.

[4] Borghaei H, Paz-Ares L, Horn L, Spigel DR, et al. Nivolumab versus docetaxel in advanced nonsquamous non-small-cell lung cancer. N Engl J Med 2015 Oct 22;373(17):1627-39.

[5] Motzer RJ, Escudier B, McDermott DF, et al. Nivolumab versus everolimus in advanced renal-cell carcinoma.

N Engl J Med 2015 Nov 5;373(19):1803-13.
[6] Raedler LA. Opdivo (Nivolumab): Second PD-1 Inhibitor Receives FDA Approval for Unresectable or Metastatic Melanoma. Am Health Drug Benefits 2015;8(Spec Feature):180-3.
[7] Kazandjian D, Suzman DL, Blumenthal G, et al. FDA Approval Summary: Nivolumab for the Treatment of Metastatic Non-Small Cell Lung Cancer With Progression On or After Platinum-Based Chemotherapy. Oncologist 2016 May;21(5):634-42.
[8] Xu JX, Maher VE, Zhang L, et al. FDA Approval Summary: Nivolumab in Advanced Renal Cell Carcinoma After Anti-Angiogenic Therapy and Exploratory Predictive Biomarker Analysis 2017 ; 22(3):311-317.
[9] Nivolumab (Opdivo) Approved in Japan for the Treatment of Advanced Melanoma. Available at: http://www.ajmc.com/newsroom/nivolumab-opdivo-approved-in-japan-for-the-treatment-of-advanced-melanoma. Accessed 15 January 2018.
[10] Curran MA, Montalvo W, Yagita H, et al. PD-1 and CTLA-4 combination blockade expands infiltrating T cells and reduces regulatory T and myeloid cells within B16 melanoma tumors. PNAS 2010;107: 4275-80.
[11] Stahl M, Walz MK, Stuschke M, et al. Phase III comparison of preoperative chemotherapy compared with chemoradiotherapy in patients with locally advanced adenocarcinoma of the esophagogastric junction. J Clin Oncol 2009;27(6):851-6.
